# Conditions under which faithful cultural transmission through teaching promotes cumulative cultural evolution

**DOI:** 10.1038/s41598-023-47018-7

**Published:** 2023-11-28

**Authors:** Seiya Nakata, Masanori Takezawa

**Affiliations:** 1 Graduate School of Humanities and Human Sciences, Hokkaido University, Sapporo, Japan; 2 Japan Society for the Promotion of Science, Tokyo, Japan; 3 Center for Experimental Research in Social Sciences, Hokkaido University, Sapporo, Japan; 4 Center for Human Nature, Artificial Intelligence and Neuroscience, Hokkaido University, Sapporo, Japan; 5 Present Address: Faculty of Humanities and Human Sciences, Hokkaido University, N10W7, Kita-ku, Sapporo, Hokkaido 060-0810 Japan

**Keywords:** Cultural evolution, Computational models

## Abstract

It has been argued that teaching promotes the accurate transmission of cultural traits and eventually leads to cumulative cultural evolution (CCE). However, previous studies have questioned this argument. In this study, we modified the action sequences model into a network exploring model with reinforcement learning to examine the conditions under which teaching promotes CCE. Our model incorporates a time trade-off between innovation and teaching. Simulations revealed that the positive influence of teaching on CCE depends on task difficulty. When the task was too difficult and advanced, such that it could not be accomplished through individual learning within a limited time, spending more time on teaching—even at the expense of time for innovation—contributed to CCE. On the contrary, the easier the task, the more time was spent on innovation than on teaching, which contributed to the improvement of performance. These findings suggest that teaching becomes more valuable as cultures become more complex. Therefore, humanity must have co-evolved a complex cumulative culture and teaching that supports cultural fidelity.

## Introduction

Recent studies have revealed that culture is ubiquitous in the animal world. Non-human animals can socially learn various techniques by observing others (e.g., Chimpanzees^1^; common chaffinches (*Fringilla coelebs*)^2^; French grunt (*Haemulon flavolineatum*)^3^; killer whale (*Orcinus orca*)^4^; bottlenose dolphin (*Tursiops sp.*)^5^; Japanese monkey (*Macaca fuscata*)^6^). However, human culture is distinct from animal cultures in its unique ability to evolve cumulatively. While chimpanzees transmit and maintain simple cultural traditions, humans continuously improve the culture transmitted from previous generations and accumulate the improvements over generations. As is summarized in the concept of “ratchet effect”^7^, human culture accumulates its complexity and utility over generations. Cumulative culture eventually attains a level that cannot be invented by a single individual within their lifetime^8^.

Researchers have investigated two critical mechanisms that produce cumulative cultural evolution (CCE): innovation and faithful transmission. Innovation is necessary to increase the utility and complexity of transmitted skills and knowledge. Some research suggests that non-human animals can also innovate^9^. Innovations play a critical role in producing adaptive behavioral variants in a population^10^. However, the faithful transmission of complex skills and knowledge seems rare among species other than humans. As culture becomes more complex, learning it socially becomes more difficult, causing the culture to potentially disappear. If a complex cultural variant is not faithfully transmitted to the succeeding generation, it becomes almost impossible to accumulate innovation onto the cultural variants transmitted from the previous generations, resulting in stagnation or even deterioration of culture^11^. Some researchers have argued that CCE is rare because only humans possess mechanisms to accurately transmit advanced complex culture to the next generation ^7,8,12,13^. One of the candidate mechanisms that sustains faithful transmission of culture is teaching^14–16^.

The importance of teaching for CCE has usually been discussed based on the results of experimental studies. However, given some discrepancies and limitations of empirical studies, a theoretical approach using computational models may be useful. Here, we developed computational models of teaching to demonstrate that a micro-level mechanism, that is, teaching promotes a macro-level phenomenon, that is, CCE.

### Does teaching contribute to cumulative cultural evolution?

Teaching behavior is quite common in human society, and humans developed institutionalized teaching in the form of a modern school system. In contrast, teaching is rarely observed among other animals^17^. Unlike the limited application among other animals, teaching among humans is widely applied in various contexts and plays a critical role in transmitting complex skills and knowledge that constitute significant parts of human culture^18,19^. Furthermore, human teaching is considered to have an ancient history. Archeological and anthropological research has shown that humans possessing proficient tool-making skills have been teaching beginners how to make tools since the age of stone tool-making in the hunter-gatherer society^20,21^. In modern times, humans’ active teaching behavior appears universally from Western societies to hunter-gatherer societies^22^. Archeological, anthropological, and historical data provide rich insights into human evolutionary history, but it is difficult to prove the causality between teaching and the emergence of CCE. Therefore, researchers have collected indirect evidence through laboratory experiments. For example, Caldwell et al. demonstrated that direct active teaching is necessary for transmitting complex knot-tying skills while simple knot-tying skills can be transmitted through emulation (i.e., observation of the end-state) or imitation (i.e., observation of intermediate states) alone^16^. Morgan et al. also showed that teaching with gestural or verbal communication is necessary for transmitting complex stone tool-making skills^23^. They further showed that, with the help of teaching, levels of complexity of tool-making did not deteriorate during transmission across multiple generations.

These studies demonstrated that active and direct teaching helps retain the transmission of complex skills and technology. Other studies have investigated whether teaching promoted the gradual improvement of inherited technologies and CCE. For example, Caldwell and Millen conducted an experiment wherein the skill required for building paper airplanes that fly the farthest was transmitted in various ways, including emulation, imitation, and teaching^24^. They did not observe any significant effect of teaching on CCE. Low task difficulty could be a possible reason for the insignificant result in their study. Building paper airplanes does not require complex technology, and such techniques could be easily identified with observation. To investigate the role of active teaching in CCE, Zwirner and Thornton asked participants to build a basket to carry as much rice as possible using 13 different materials, including strings, skewers, and drawing pins^25^. This task is more complex than building paper airplanes as there are various ways to combine materials to build a basket. The researchers found that, compared to imitation and emulation, teaching increases the mass of rice carried by the basket over generations. This result suggests that teaching promotes CCE.

### Computational model of teaching and cumulative cultural evolution

Mathematical models allow us to examine theoretical hypotheses more rigorously than laboratory experiments. It is difficult to bring the complex products of CCE, such as modern technology and scientific knowledge, into the laboratory. Formalized models can be used in a complementary way to test theoretical explanations by manipulating important parameters, such as task difficulty. Several researchers have proposed mathematical models of teaching and CCE^26,27^. These models postulate that as a skill gets more difficult, it becomes increasingly difficult for a child to acquire it. Thus, teaching is defined as a costly behavioral phenotype of adults that helps children acquire complex advanced skills from a previous generation. These theoretical models demonstrated that cumulative knowledge and teaching co-evolve under certain situations. The models assumed a priori that teaching ensures the faithful transmission of complex knowledge and aimed to clarify theoretical relationships such as whether the biological cost of teaching is worth it. From a cognitive perspective, cultural transmission (whether teaching or observing) is not a process of directly copying information onto the brain. The cultural information inside an individual’s mind generates an observable behavior. Then, an observer acquires the information that produces similar behaviors^28^. The mechanism through which teaching promotes accurate transmission depends on the task and learning process. In addition, there are various forms of teaching, such as politely displaying each step of the action or attracting the learner's attention by using gestures and eye contact (cf. Csibra and Gergely^18^). Transmission fidelity is not an intrinsic property of the social learning mechanism^29^. Consequently, it is necessary to build a new computational model that expresses the process of learning and teaching of technology. Such a model would allow us to elaborate on the various arguments from laboratory experiments, such as the relationship between faithful transmission and CCE^30^.

The process of skill acquisition is formulated using reinforcement learning models that are used in a wide range of fields, including machine learning, computational neuroscience, and cognitive science. Some researchers took a similar approach and formulated computational models of social learning based on reinforcement learning in the Markov decision processes^31,32^. Our study formulated computational processes of teaching based on reinforcement learning and investigated how teaching promotes the cumulative evolution of complex technology. We defined skill acquisition as learning effective behavior sequences and modeled the process as an exploration problem in a huge network. Through this new computational model, we demonstrate that the accumulation of micro-processes of learning and teaching creates a macro phenomenon, that is, CCE of advanced technology. We also use this computational model to examine two issues that have not been fully explored in empirical studies: task difficulty and a time trade-off between innovation and teaching.

## Models

### Modeling the learning of complex techniques as a multi-goal network

The process of learning complex techniques, such as hunting and producing tools, involves arranging various action steps in order. Researchers have modeled the learning process as reinforcement learning of action sequences^31–33^. We modified the action sequence model into a complex multi-goal network to express the acquisition of more complex technologies. In the network, nodes represent states, and edges between nodes represent possible actions. When agents choose an action, they transition to an adjacent state. Edges are bi-directional, and the agent can move around the network until they reach a goal node. If agents reach a goal, they receive a reward. The reward is represented by the square of the shortest distance from the start node, because the larger reward gaps between goals make the learning fast and reduce the computation time. In the Supplementary Information, we confirmed the difficulty of earning large rewards with the multi-goal network.

We adopted a reinforcement learning model of action sequences used in Enquist et al.^33^. Their model is based on a computationally simple theory of conditioned reinforcement and is biologically valid as a learning model for action sequences. In the first round, the agents move randomly between the states because they have no information about the network. They receive rewards once the agents reach a goal state, and the immediately preceding action and state are reinforced (see Supplementary Information for more details).

### The model of teaching

We modeled teaching as following the action sequence step by step. A teacher gets a student (a next generation agent) to trace the path they took in the final round (i.e., a quasi-optimal path) for *T* rounds. If the teacher took the path state1 → state2 → state3 (goal) in the final round, the student is taught to follow the same path state1 → state2 → state3 (goal) for *T* rounds. In the first teaching, the student updates the value of a state just before the goal (state2) and an action to reach the goal (state2 → state3); in the second teaching, the value of the same state and action is further updated, plus another previous state (state1) and action (state1 → state2). By repeating this process, the student becomes more likely to follow the same path as the teacher when exploring in individual learning. It should be noted that teaching is not a perfect copy. For instance, 10,000 students were taught the shortest path to a goal of distance 5 in the network shown in Fig. S1; after being taught the path for one round (i.e., $T = 1$), only 5.75% arrived at the goal in the following round. Due to the nature of the reinforcement learning algorithm, students learn more accurately when teaching involves more rounds (*T*). However, since the agent's choice of action is probabilistic, a completely accurate copy cannot be guaranteed even if students are taught for a longer time. Although our teaching model may not apply to all real-life teaching scenarios. we assume a form of careful teaching intending to faithfully transmit complex techniques.

The path teachers took in the final round is selected probabilistically with the SoftMax rule. In the Supplementary Information, we examined greedy teachers who produce the path to be taught in the next generation by always selecting actions having larger Q values (which is analogous to applying the SoftMax rule with the positive infinite inverse temperature). Interestingly, CCE was less likely to occur under the greedy teacher model compared to the probabilistic teacher model. In the Results section, we report only the results of the probabilistic teacher model and, in the Discussion section, consider why the current model outperforms the greedy teacher model.

Previous studies have formulated mathematical models of social learning that involve direct learning of very short action sequences observed by naive learners (e.g., Lind et al.^32^). Our model is distinct from such models of other social learning. Looking at our model as observational social learning, once agents have observed the process, they must memorize and reinforce all paths (actions) to the goal. However, if the number of steps to the goal is very large, it becomes impossible to learn the action sequence by observation alone. In actual simulations (described later), the paths taken by the agent involved several hundred steps in some cases. Thus, careful step-by-step teaching is necessary rather than observation and memorization to follow such long paths without error. Furthermore, from the point of view of teacher behavior change, our model is considered as teaching, not other social learning^34^. In the teaching phase, teacher agents stop learning and repeatedly show the same action sequence (quasi-optimal solution learned by the teacher) to the student. Thus, teachers change their behavior to promote student learning.

Our model can be clearly linked to the basic framework of cumulative cultural evolution^35^. Individual learning exploration leads to a change in behavior. Teaching transfers behavior to other individuals. With learned behavior, agents acquire higher rewards, which is a proxy for genetic and/or cultural fitness. These processes are then repeated across generations.

### Trade-off between teaching (faithful transmission) and individual learning (innovation)

Complex cumulative cultures often comprise considerable amounts of information, and the faithful transmission of complex cultures requires longer periods of teaching^36^. However, individuals have limited time available. If learners spend too much time being taught for cultural fidelity, they are likely to lose the opportunity to innovate through individual learning. In other words, there is a time trade-off relationship between innovation through individual learning and faithful transmission through teaching, both of which are essential for CCE. Ignoring this trade-off would lead to an overestimation of the impact of teaching on CCE.

**Figure 1:**
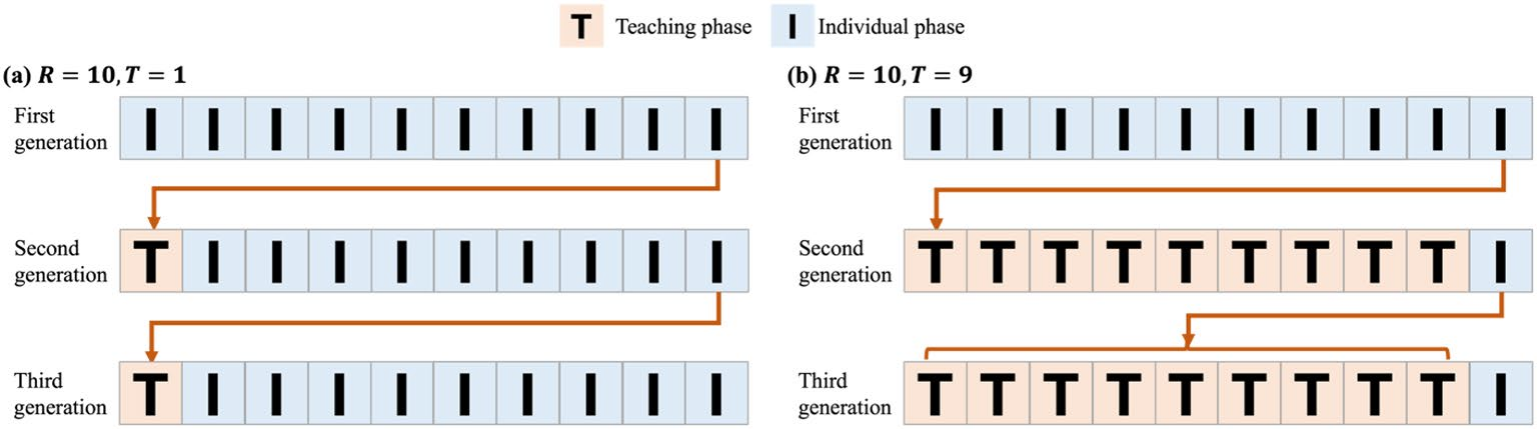
A conceptual diagram of the time trade-off between individual learning and teaching. First generation agents learn individually for all rounds. After the second generation, for the first T rounds, agents are taught to follow the path taken by agents of the previous generation in the final round. (**a**) The case where *R* = 10, *T* = 1. When agents spend only 1 round in the teaching phase, they have more opportunities to innovate instead of inaccurately transmitting. (**b**) The case where *R* = 10, *T* = 9. When agents spend 9 rounds in the teaching phase, they are more likely to reproduce the same action sequences of the previous generation, instead of having fewer opportunities to innovate.

Therefore, we introduced a time trade-off between innovation (individual learning) and faithful transmission (teaching). Figure 1 represents a conceptual diagram of this trade-off. Parameter *R* is the number of rounds that agents can spend on learning per generation. Agents of the second generation spend the first *T* rounds in the teaching phase and the remaining *R-T* rounds in the individual learning phase. If the teaching phase is longer, the agent can learn a path from the teacher (the agent of the first generation) more faithfully. However, when *R*-*T* becomes shorter, the agent of the second generation loses opportunities to explore other goals with higher rewards.

## Results

### Difficulty of the task: the simulation of individual learning using the multi-goal network

**Figure 2:**
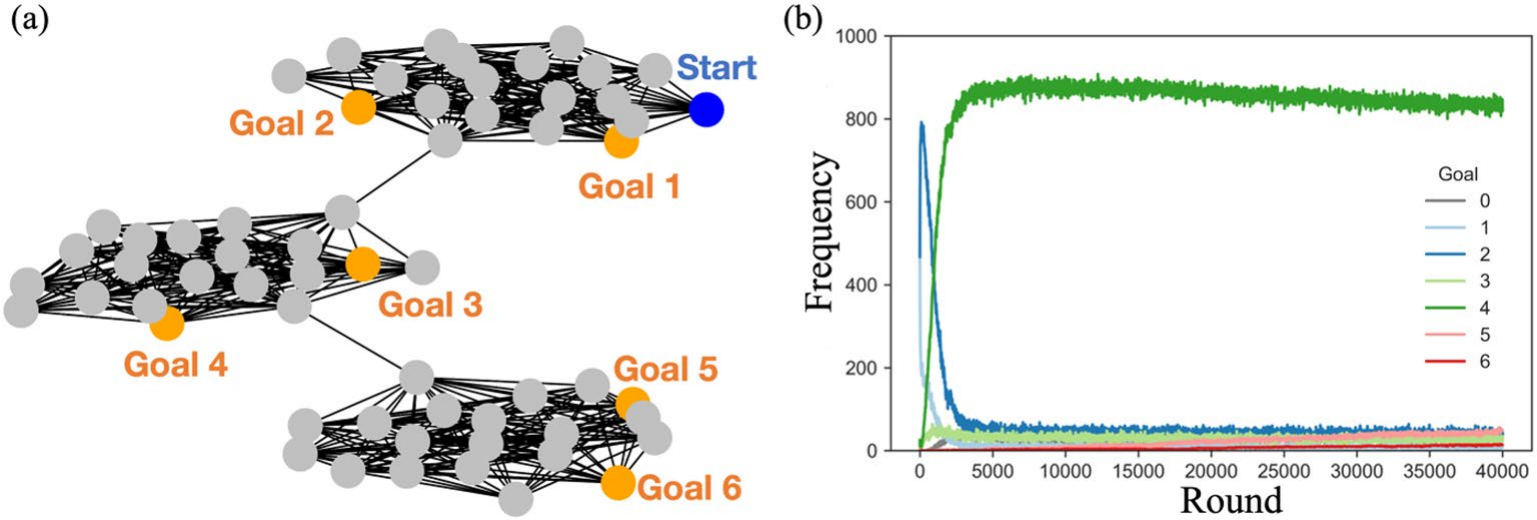
(**a**) An undirected multi-goal network containing three random sub-networks connected in series. The six nodes at the shortest distance from the start node, from 1 to 6, were set as goals. The reward for reaching each goal was set to be the square of the shortest distance. (**b**) Frequency of the agents who reached each goal among 40,000 rounds of individual learning ($\alpha = 0.9, \beta = 0.5$). Goal 0 indicates the frequency of agents who failed to reach the goal within the upper limit of 1000 steps.

We use a network comprising three clusters (Fig. 2a). We constructed three random undirected networks of 20 nodes using the Erdős–Rényi model and connected them linearly^37^. Inspired by the task design with a multi-modal adaptive landscape^38,39^, we designed this type of structure to make it more difficult for agents to reach distant goals. To test the difficulty of the task, we ran simulations where agents explored the network for only one round (i.e., random exploration). Consequently, 96% of the agents reached goal 1 or 2, and none of them reached goals 5 and 6 (see Supplementary Information for more details).

**Figure 3:**
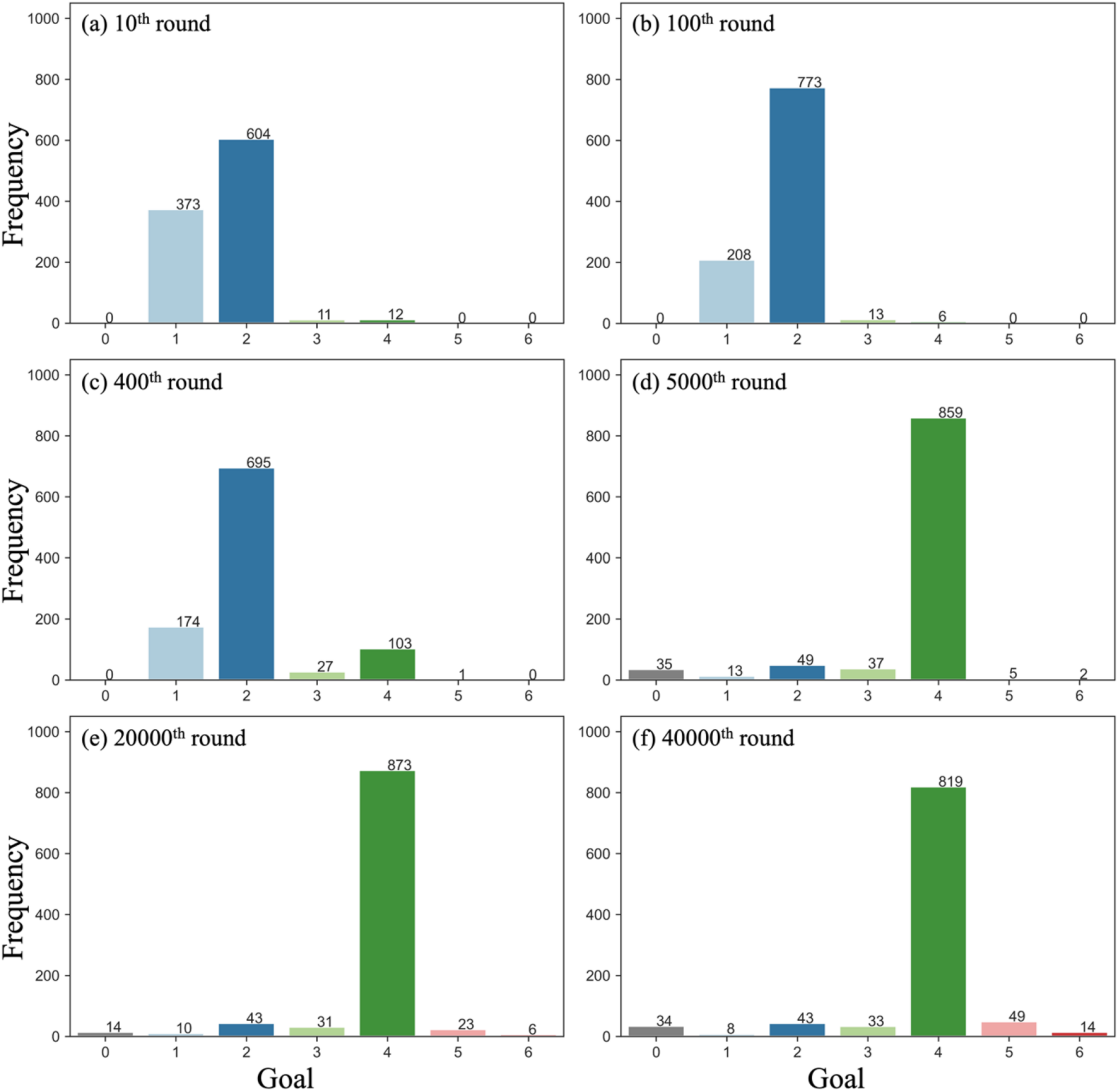
Frequency of agents reaching each goal in a particular round of individual learning ($\alpha = 0.9, \beta = 0.5$). Goal 0 indicates the frequency of agents who failed to reach the goal within the upper limit of 1000 steps.

Even when the reinforcement learning algorithm allowed the agent to learn beneficial behaviors with higher rewards, it took a significant number of rounds to reach the distant goal. Figure 2b shows the frequency of agents reaching each goal when 1,000 agents explored the network for 40,000 rounds with individual learning. As mentioned earlier, most agents could only reach goals 1 or 2 in the first round. Repeating the exploration process many times through reinforcement learning increases the proportion of agents who can reach farther goals. After more than 400 rounds of exploration, about 70% of the agents reached goal 2 (Fig. 3c). With further exploration, the number of agents reaching goal 4 gradually increased, and almost all agents reached goal 4 in the 5000th round (Fig. 3d). Even after increasing the number of rounds, few agents reached goals 5 or 6 (Fig. 3e,f). Thus, it was very difficult to obtain high rewards in our multi-goal network.

In the Supplementary Information, we replicated all the simulations with a single-cluster network, which facilitates agents to more easily reach distant goals than the three-cluster network. We confirmed that the qualitative results reported in the next section remain unchanged.

### The effect of teaching on cumulative cultural evolution

Cumulative culture is often described as a complex and sophisticated culture that a single individual cannot invent. This has two possible interpretations. First, an individual cannot invent it because it is theoretically impossible. Second, an individual can invent it theoretically; however, it is practically impossible because of time limitations. Many CCE studies have adopted the second interpretation as the definition of the CCE^40–43^. We employed the latter definition. We compared the mean performance achieved in each generation with the mean performance of agents who explore alone within the fixed time. If the former exceeds the latter, we call it CCE. We also examined CCE based on the first interpretation by comparing it with the performance of immortal agents who could explore the task for a very long time.

Following the above discussion, task difficulty was operationalized by manipulating the amount of time an individual can engage. The difficulty of a task may be considered as an intrinsic property of the task^44^. Therefore, one possible way to manipulate task difficulty is to fix the time and vary the network’s size and density. However, task difficulty is also affected by the amount of time an individual can spend on it. Indeed, in many experiments, participants improved their performance in proportion to the time they spent (e.g., virtual arrowhead design^38^; basket production from a predetermined list of materials^25^; paper and pipe-cleaner tools manufacture^44^). Our method of manipulating task difficulty according to the time (parameter *R*) is advantageous over the manipulation of task structure. Changing the network’s size or density qualitatively alters task properties and may hinder the examination of the continuous nature of task difficulty, which is described in Figs. 2b and 3.

Further, we examined the extent to which accurate transmission facilitates CCE. In our model, there is a time trade-off between teaching for accurate transmission and individual learning for innovation. We ran several simulations with varied ratios of teaching to individual learning to investigate whether spending more time on teaching would promote CCE, even if the opportunity for innovation is lost.

**Figure 4:**
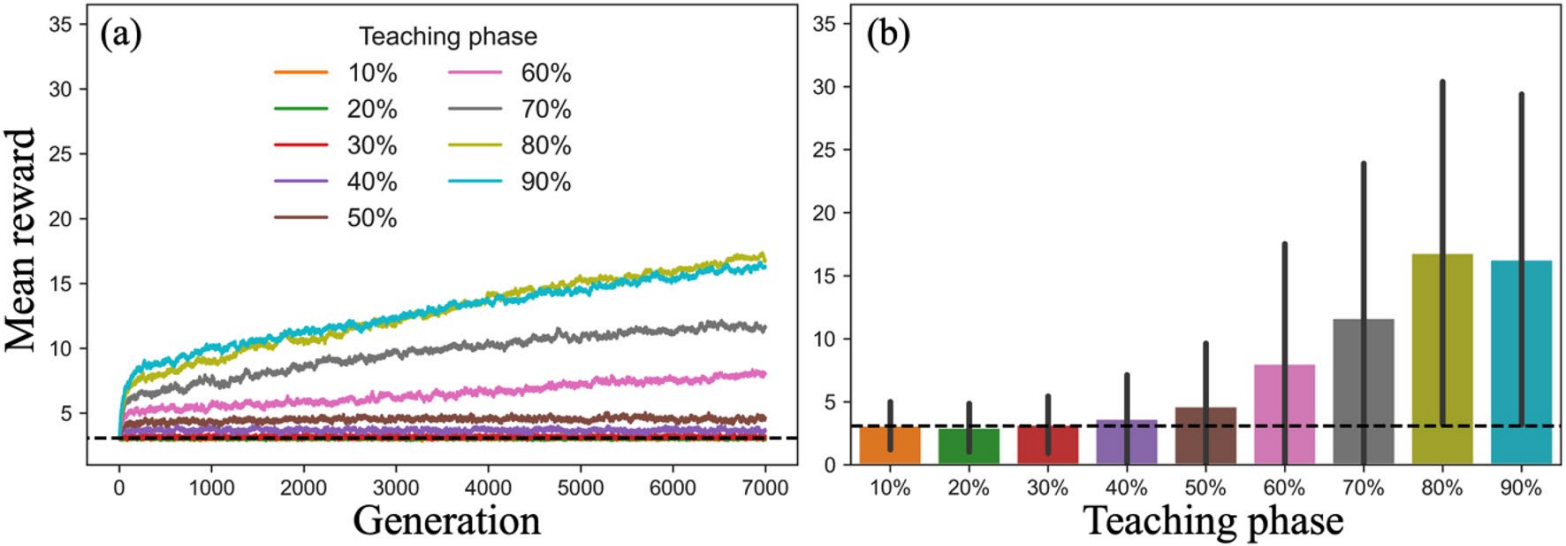
Mean reward for each length of teaching phase (*R* = 10, $\alpha = 0.9, \beta = 0.5$). (**a**) Colored lines represent the mean reward (*n* = 1000) at the final round of each generation in each length of teaching phase. (**b**) Bars represent mean reward of 1000 agents in the 7000th generation for each teaching phase. The error bars represent standard deviation of reward. The dashed lines in both figures show the mean reward acquired through individual learning alone for 10 rounds as the reference line.

In this section, we show only the results of simulations with *R* = 10 (we will change the value of *R* in the next section.). This is the minimum value of the parameter *R* in this study. In other words, it is the most difficult setting for agents to achieve high performance within their limited time. We varied the length of the teaching phase from 10 to 90% in 10% increments over 10 rounds (i.e., one to nine rounds). We ran 1000 simulations with 7000 generations for each length of the teaching phase. Figure 4a depicts the mean reward for each length of teaching phase over generations. The minimum reward is 1 (goal 1). The maximum reward is 36 (goal 6). Each data point represents a mean reward of 1000 agents in the final round of each generation. The dashed lines represent the mean reward (= 3.43) of 1000 agents who performed 10 rounds of individual learning as the reference line. Mean reward above the reference line indicates CCE.

The mean reward is far from the reference line as the generation increases, except in the case where the length of the teaching phase is 10–30% (Fig. 4a). In other words, teaching among generations causes CCE except when the length of the teaching phase is 10–30%, wherein the mean reward is almost the same as the reference line even as the generation progresses; that is, agents are stuck in the first cluster with goals 1 or 2, and the level of technology remains stagnant.

We found that long-term teaching promotes the speed of CCE. Figure 4b represents the mean reward by the length of teaching phase in the 7000th generation. We observed a correlation between the length of the teaching phase and the mean reward (Fig. 4b).

### The effect of task difficulty (the length of time spent by agents)

In the model, a longer period of teaching facilitates more accurate transmission. Given the findings of previous studies, long-term teaching contributes to CCE, especially when the technology is complex and difficult to acquire within the time individuals can engage. As discussed above, task difficulty is determined by the length of time the agent can spend on it. If an agent has sufficient time, they would be able to achieve a high performance without any teaching. Thus, by increasing the value of parameter *R*, task difficulty for the agent decreases and vice versa. To clarify the impact of task difficulty, we ran simulations with varied values of *R*, that is, 100, 400, and 5,000. Total lengths of generations varied for each simulation due to the hardware limitations of running simulations; however, we ran simulations until the effects of teaching became clear. The length of the teaching phase was set as 10–90% in 10% increments with respect to *R*. That is, if *R* = 100, the length of the teaching phase is 10, 20, 30, … 80, 90.

**Figure 5:**
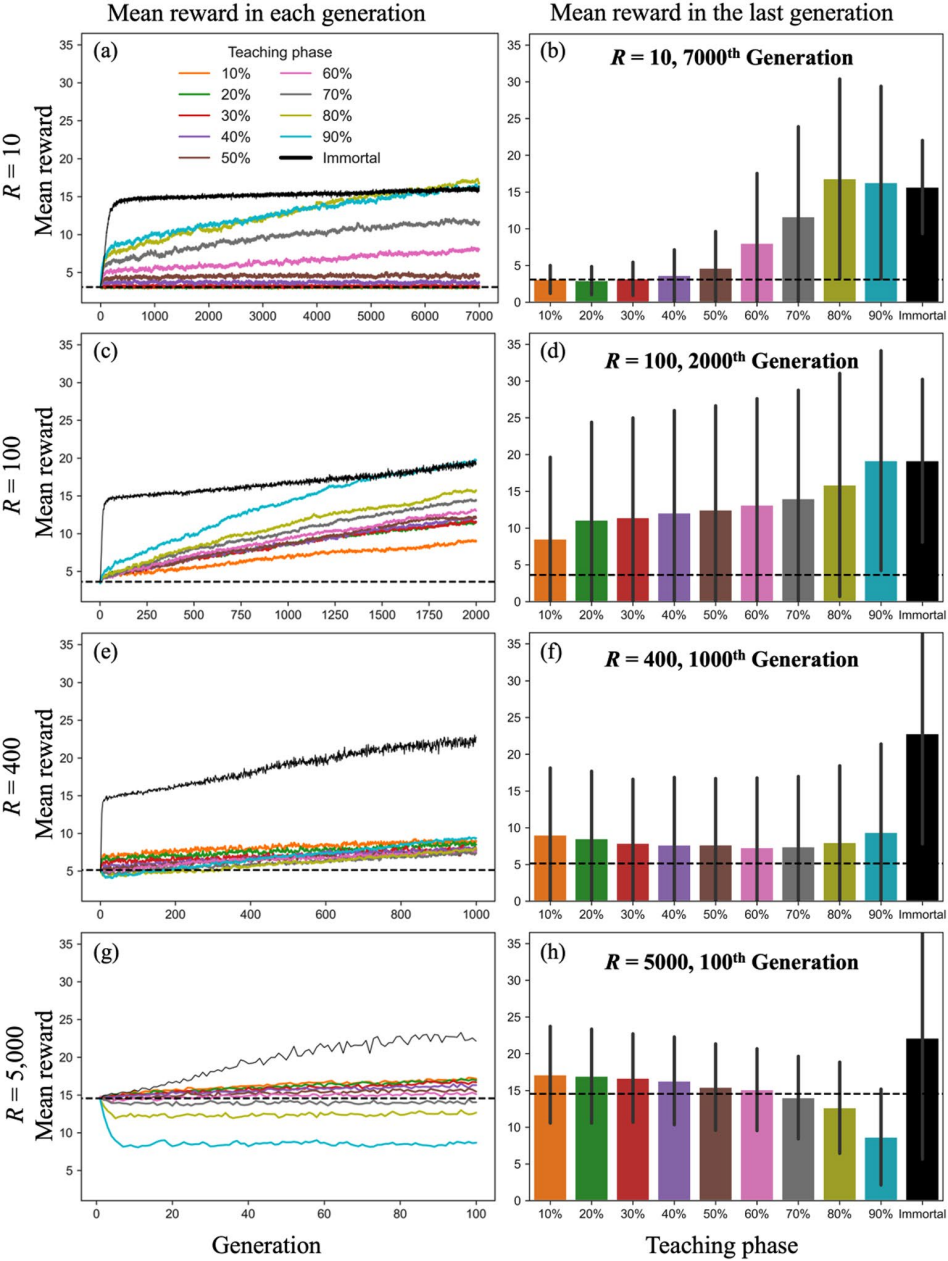
Comparison of the performance of immortal agents with that of agents with teaching ($\alpha = 0.9, \beta = 0.5$). Left column: The solid black lines represent the mean reward of 1,000 agents who have performed only individual learning corresponding with the cumulative number of rounds of the simulation with teaching. The line is plotted per each *R* rounds; 10 rounds for *R* = 10, 100 rounds for *R* = 100, and so on. Colored lines show the mean reward in the final round of each generation for each length of teaching phase. Right column: Colored bars show the mean reward in the last generation for the length of teaching phase, and the black bar shows the mean reward of immortal agents in the final round. Error bars show the standard deviation. The dashed lines in all figures show mean reward acquired through individual learning alone for *R* rounds.

First, as well as the simulation with *R* = 10 (Fig. 5a,b), CCE occurs except for a few conditions. However, task difficulty (the value of *R*) determines the effect of teaching length on CCE. In the simulation with *R* = 100, the condition where the task is easier for the agent than at *R* = 10, we find the positive relationship between the length of teaching performance again (Fig. 5d). In the simulation with *R* = 400, wherein the task is even easier, the positive correlation between length of teaching and mean reward disappears (Fig. 5f). In the simulation with *R* = 5,000, the relationship between length of teaching and performance is reversed, showing a negative correlation (Fig. 5h). When *R* is 5000 and the length of teaching phases is above 70%, the performance of taught agents was below the performance of an individual who explored solely through individual learning within the duration of the same time (dashed line). In other words, long-term teaching deprives agents of the opportunity to explore and reduces their performance in situations where agents can easily achieve high performance in their limited time.

### Comparison with the performance of no loss of information

A certain amount of information will be lost when technologies and knowledge are transmitted from individual to individual. The loss of information becomes more noticeable as the culture to be transmitted becomes more complex; this deteriorates transmission fidelity. The role of teaching may be to reduce information loss and preserve the complex culture in the course of cultural transmission. To examine such effects of teaching, we measured the performance of immortal individuals who continued individual learning alone for several thousands of generations, wherein learned information was never lost. The solid black lines in Fig. 5a,c,e,g represent the mean reward of 1,000 immortal agents who performed individual learning for the rounds corresponding with the number of previous simulations with the cultural transmission. Comparisons with immortal agents also show whether the first type of CCE mentioned above has occurred in the current simulations. The results show that immortal agents without information loss improved their performance much faster than agents who received teaching. However, when the task was relatively difficult (*R* = 10 and 100, Fig. 5a,c), mortal agents could catch up to and exceed the performance of immortal agents over generations if the teaching durations were long enough.

## Discussion

It has been argued that faithful transmission through teaching is the key to CCE, but previous studies have reported inconsistent conclusions. Using computer simulation, we examined the conditions under which faithful transmission through teaching promotes CCE. We built a computational model that incorporates the following two elements: goals with higher rewards are more difficult to reach and a time trade-off between innovation through individual learning and faithful transmission through teaching. In addition, since previous studies have suggested that task difficulty is a critical factor, we controlled the relative task difficulty by varying the number of rounds per generation.

To summarize the results of our research, the effect of faithful transmission through long-term teaching on CCE depends on the number of rounds per generation (*R*). When *R* is low, long-term teaching promotes CCE even though agents lose opportunities for innovation (i.e., individual learning). In contrast, when *R* is high, long-term teaching is less effective. Instead, spending more time on individual learning for innovation contributes to CCE. Our findings suggest that the value of teaching that promotes faithful cultural transmission is high in an environment where complex technologies exist. It may also indicate the effectiveness of long-term teaching in an environment where there are so many types of skills to learn. In our model, the agent had only one task to learn, but individuals who learn more diverse skills are likely to have an advantage. Even if the total time spent learning each skill is reduced, they would likely be able to acquire more skills efficiently by prioritizing expert instruction. In the Supplementary Information, we have confirmed that a series of simulation results can be reproduced with different parameters of reinforcement learning and a different network structure of task.

We also examined situations where there was no trade-off between accurate transmission and innovation. When agents had infinite time and no information loss, their performance improved rapidly. However, immortal agents did not always outperform those that transmitted incomplete information through teaching, especially when the task was difficult. In other words, teaching caused a more strictly defined CCE. Our results emphasize that the task difficulty is important for understanding CCE, and examining the effect of task properties would be an important theoretical contribution to CCE of technology in general. For example, large average path lengths and average degrees would make the exploration more difficult. Investigating how their interaction would change the form of transmission required for CCE is a promising direction.

Focusing on task difficulty helps us understand the results of existing experimental studies. When the tasks of previous studies are not complex, the conclusion is likely to be drawn that teaching and high-fidelity transmission are not necessary for CCE^24,45,46^. On the other hand, experimental studies showing the usefulness of teaching used more complex tasks^25,47^. Relationships between teaching and complex technologies may be bi-directional: while our study showed that high-fidelity teaching promotes the CCE of complex technologies, Lucas et al.^44^ and Montrey and Shultz^47^ suggested that complex technologies drive the evolution of high-fidelity teaching.

We considered task difficulty in terms of how much reward could be achieved in a limited amount of time and attempted to control for relative difficulty by the number of rounds. On the other hand, other studies using the NK model have controlled for task difficulty more directly and have successfully examined the relationship with performance^48,49^. Our model may also be able to control task difficulty independent of the number of rounds. For example, the effect of network size and density on the goals that agents can achieve should be systematically investigated in large-scale studies, either by simulation or by laboratory experiments with human participants. It would also be possible to investigate whether a more difficult network would produce similar results to those obtained with a shorter number of rounds.

In the Supplementary Information, we examined greedy teachers who teach paths that fully exploit their experience by consistently selecting a path with the highest Q values. CCE was rarely observed under the greedy teacher model, however. Contrasts between the two models may indicate the importance of probabilistic fluctuations caused by the teacher’s exploration. The paths taught to students by a probabilistic teacher contained many redundant paths, rather than the shortest path to the goal. Such redundancy may promote students’ exploration in individual phases. It has been argued that more explicit and direct teaching (high fidelity transmission), which strongly constrains children's behavior, may inhibit exploration and innovation^15^. Teaching with redundancy may help escape from such constraints caused by faithful teaching.

We analyzed the effect of the length of the teaching phase on CCE and found positive or negative correlations in some conditions. However, varying the length of the teaching phase in one-round increments may produce inverted U-shaped relationships. For example, in a simulation with *R* = 100, the mean reward was maximum at the 90% teaching phase but will decrease if the teaching phase is extended to 99 rounds. Similarly, in the simulation with *R* = 5,000, the mean reward was maximum at a 10% teaching phase but will decrease if the teaching phase is only one round.

While most studies of cultural evolution use black-box models of the processes of learning and transmission of culture, we constructed a model that assumes a cognitive agent. Miton and DeDeo similarly constructed a model based on statistical physics that takes into account the cognitive processes of learning and teaching^50^. Both studies suggest the importance of both individual and social learning in cultural transmission. On the other hand, their model showed that teaching only a few key features can achieve accurate transmission. In our model, if the teacher can selectively teach important information, the student can achieve the same goal as the teacher with a shorter teaching phase. For example, if the teacher teaches the shortest path to the goal instead of the redundant path, accurate transmission is easier to achieve in a short teaching phase. In addition, Miton and DeDeo analyzed accuracy using Hamming distance. Our model could also analyze the relationship between teaching and fidelity by measuring the distance between the action sequence transmitted by a teacher and the action sequence finally learned by the agent. Cognitive models such as ours and Miton's may open the way to directly study the relationship between transmission fidelity and cumulative cultural evolution.

Researchers have shown that a type of cumulative cultural variation is observed among non-human animals. A cumulative cultural optimization in a fixed solution, which cannot expand the problem space^28^, has been observed among non-human animals^45,51^. Mesoudi and Thornton^35^ and Derex^52^ argued that the uniqueness of CCE among humans is open-endedness. Our model represents the discovery of new cultural traits, but it is still a kind of cumulative cultural optimization because the number of goals in our task was finite. Open-ended CCE is worthy of investigation^53^, and it is an important avenue for further testing the impact of teaching on open-ended CCE. To study open-ended CCE, it may be necessary to consider not only a one-to-one model of cultural transmission, as in this study, but also a larger group model of cultural transmission. Some studies have found that a large population size is necessary for CCE^11,54,55^. Given the large population size, information is transmitted not only from parents (vertical transmission) but also from other unrelated adults (oblique transmission) and people in the same age group (horizontal transmission). In addition, complex interactions such as population size, structure, and transmission mechanisms have been shown to influence CCE^56–58^. These allow the population to innovate and combine various traits, thus future research should incorporate these interactions into the model.

### Supplementary Information

Supplementary Information.

## Data Availability

All simulation codes are available at https://github.com/SeiyaNAKATA/ABM_TeachingAndCCE.git.

## References

[1] Whiten A (1999). Cultures in chimpanzees. Nature.

[2] Lynch A, Baker AJ (1993). A population memetics approach to cultural evolution in Chaffinch song: Meme diversity within populations. Am. Nat..

[3] Helfman GS, Schultz ET (1984). Social transmission of behavioural traditions in a coral reef fish. Anim. Behav..

[4] Ford JKB (1991). Vocal traditions among resident killer whales (*Orcinus orca*) in coastal waters of British Columbia. Can. J. Zool..

[5] Krützen M (2005). Cultural transmission of tool use in bottlenose dolphins. Proc. Natl. Acad. Sci..

[6] Schofield DP, McGrew WC, Takahashi A, Hirata S (2018). Cumulative culture in nonhumans: Overlooked findings from Japanese monkeys?. Primates.

[7] Tennie C, Call J, Tomasello M (2009). Ratcheting up the ratchet: On the evolution of cumulative culture. Philos. Trans. Biol. Sci..

[8] Boyd, R. & Richerson, P. J. Why culture is common, but cultural evolution is rare. In *Evolution of Social Behaviour Patterns in Primates and Man* 77–93 (Oxford University Press, 1996).

[9] Mesoudi A, Richerson PJ, Christiansen MH (2013). The cultural evolution of technology and science. Cultural Evolution: Society, Technology, Language, and Religion.

[10] Reader SM, Morand-Ferron J, Flynn E (2016). Animal and human innovation: novel problems and novel solutions. Philos. Trans. R. Soc. B Biol. Sci..

[11] Henrich J (2004). Demography and cultural evolution: How adaptive cultural processes can produce maladaptive losses—The Tasmanian case. Am. Antiq..

[12] Tomasello M (1999). The Cultural Origins of Human Cognition.

[13] Lewis HM, Laland KN (2012). Transmission fidelity is the key to the build-up of cumulative culture. Philos. Trans. R. Soc. B Biol. Sci..

[14] Mesoudi, A. Cultural evolution: How Darwinian theory can explain human culture and synthesize the social sciences. In *Cultural Evolution* (University of Chicago Press, 2011). 10.7208/9780226520452.

[15] Burdett ERR, Dean LG, Ronfard S (2018). A diverse and flexible teaching toolkit facilitates the human capacity for cumulative culture. Rev. Philos. Psychol..

[16] Caldwell CA, Renner E, Atkinson M (2018). Human teaching and cumulative cultural evolution. Rev. Philos. Psychol..

[17] Thornton A, Raihani NJ (2008). The evolution of teaching. Anim. Behav..

[18] Csibra G, Gergely G (2009). Natural pedagogy. Trends Cogn. Sci..

[19] Csibra G, Gergely G (2011). Natural pedagogy as evolutionary adaptation. Philos. Trans. R. Soc. B Biol. Sci..

[20] Tehrani JJ, Riede F (2008). Towards an archaeology of pedagogy: learning, teaching and the generation of material culture traditions. World Archaeol..

[21] Gärdenfors P, Högberg A (2017). The archaeology of teaching and the evolution of *Homo docens*. Curr. Anthropol..

[22] Lew-Levy S, Boyette AH (2018). Evidence for the adaptive learning function of work and work-themed play among Aka forager and Ngandu farmer children from the Congo Basin. Hum. Nat..

[23] Morgan TJH (2015). Experimental evidence for the co-evolution of hominin tool-making teaching and language. Nat. Commun..

[24] Caldwell CA, Millen AE (2009). Social learning mechanisms and cumulative cultural evolution: Is imitation necessary?. Psychol. Sci..

[25] Zwirner E, Thornton A (2015). Cognitive requirements of cumulative culture: Teaching is useful but not essential. Sci. Rep..

[26] Fogarty L, Strimling P, Laland KN (2011). The evolution of teaching: The evolution of teaching. Evolution.

[27] Castro L, Toro MA (2014). Cumulative cultural evolution: The role of teaching. J. Theor. Biol..

[28] Winters J (2019). Escaping optimization traps: The role of cultural adaptation and cultural exaptation in facilitating open-ended cumulative dynamics. Palgrave Commun..

[29] Charbonneau M (2020). Understanding cultural fidelity. Br. J. Philos. Sci..

[30] Heyes C (2018). Enquire within: cultural evolution and cognitive science. Philos. Trans. R. Soc. B Biol. Sci..

[31] Whalen A, Cownden D, Laland K (2015). The learning of action sequences through social transmission. Anim. Cogn..

[32] Lind J, Ghirlanda S, Enquist M (2019). Social learning through associative processes: A computational theory. R. Soc. Open Sci..

[33] Enquist M, Lind J, Ghirlanda S (2016). The power of associative learning and the ontogeny of optimal behaviour. R. Soc. Open Sci..

[34] Caro TM, Hauser MD (1992). Is there teaching in nonhuman animals?. Q. Rev. Biol..

[35] Mesoudi A, Thornton A (2018). What is cumulative cultural evolution?. Proc. R. Soc. B Biol. Sci..

[36] Mesoudi A (2011). Variable cultural acquisition costs constrain cumulative cultural evolution. PLoS ONE.

[37] Erdös P, Rényi A (1959). On random graphs I. Publ. Math. Debr..

[38] Mesoudi A (2008). An experimental simulation of the “copy-successful-individuals” cultural learning strategy: Adaptive landscapes, producer–scrounger dynamics, and informational access costs. Evol. Hum. Behav..

[39] Mesoudi A, O’Brien MJ (2008). The cultural transmission of great basin projectile-point technology I: An experimental simulation. Am. Antiq..

[40] Caldwell CA, Millen AE (2008). Experimental models for testing hypotheses about cumulative cultural evolution. Evol. Hum. Behav..

[41] Kempe M, Mesoudi A (2014). Experimental and theoretical models of human cultural evolution: Models of cultural evolution. Wiley Interdiscip. Rev. Cogn. Sci..

[42] Muthukrishna M, Henrich J (2016). Innovation in the collective brain. Philos. Trans. R. Soc. B Biol. Sci..

[43] Derex M, Bonnefon J-F, Boyd R, Mesoudi A (2019). Causal understanding is not necessary for the improvement of culturally evolving technology. Nat. Hum. Behav..

[44] Lucas AJ (2020). The value of teaching increases with tool complexity in cumulative cultural evolution. Proc. R. Soc. B Biol. Sci..

[45] Saldana C, Fagot J, Kirby S, Smith K, Claidière N (2019). High-fidelity copying is not necessarily the key to cumulative cultural evolution: A study in monkeys and children. Proc. R. Soc. B Biol. Sci..

[46] Reindl E, Apperly IA, Beck SR, Tennie C (2017). Young children copy cumulative technological design in the absence of action information. Sci. Rep..

[47] Montrey M, Shultz TR (2020). The evolution of high-fidelity social learning. Proc. R. Soc. B Biol. Sci..

[48] Yahosseini KS, Moussaïd M (2020). Comparing groups of independent solvers and transmission chains as methods for collective problem-solving. Sci. Rep..

[49] Boroomand A, Smaldino PE (2021). Hard work, risk-taking, and diversity in a model of collective problem solving. J. Artif. Soc. Soc. Simul..

[50] Miton H, DeDeo S (2022). The cultural transmission of tacit knowledge. J. R. Soc. Interface.

[51] Sasaki T, Biro D (2017). Cumulative culture can emerge from collective intelligence in animal groups. Nat. Commun..

[52] Derex M (2022). Human cumulative culture and the exploitation of natural phenomena. Philos. Trans. R. Soc. B Biol. Sci..

[53] Borg JM (2023). Evolved open-endedness in cultural evolution: A new dimension in open-ended evolution research. Artif. Life.

[54] Derex M, Beugin M-P, Godelle B, Raymond M (2013). Experimental evidence for the influence of group size on cultural complexity. Nature.

[55] Muthukrishna M, Shulman BW, Vasilescu V, Henrich J (2014). Sociality influences cultural complexity. Proc. R. Soc. B Biol. Sci..

[56] Cantor M (2021). Social network architecture and the tempo of cumulative cultural evolution. Proc. R. Soc. B Biol. Sci..

[57] Deffner D, Kandler A, Fogarty L (2022). Effective population size for culturally evolving traits. PLOS Comput. Biol..

[58] Derex M, Mesoudi A (2020). Cumulative cultural evolution within evolving population structures. Trends Cogn. Sci..

