# Supplementary material for: Conditions under which faithful cultural transmission through teaching promotes cumulative cultural evolution
Source: Sci Rep. 2023 Nov 28;13:20986. doi: 10.1038/s41598-023-47018-7 (PMC10684533; doi:10.1038/s41598-023-47018-7)
Supplement: Supplementary file 1 — Supplementary Information. [file 41598_2023_47018_MOESM1_ESM.docx]

Supplementary Information:

Conditions under which faithful cultural transmission through teaching promotes cumulative cultural evolution

Seiya Nakata^1,2^ & Masanori Takezawa^1,3,4^

^1^ Department of Behavioral Science, Graduate School of Letters, Hokkaido University, Japan

^2^ Japan Society for the Promotion of Science

^3^ Center for Experimental Research in Social Sciences, Hokkaido University, Japan

^4^ Center for Human Nature, Artificial Intelligence and Neuroscience, Hokkaido University, Japan

## **A, Details of the reinforcement learning model**

Reinforcement learning is a model wherein agents learn the association between action and reward. We extended the model proposed by Enquist et al. [33] to express the acquisition of very complex and advanced technology as a search task in a random network. Reinforcement learning models have two parameters: the learning rate, which adjusts learning speed, and the inverse temperature, which weights the propensity of exploration-exploitation. In the current study, we searched a set of parameters achieving almost the highest individual learning performance and decided to use the parameter values in the main text ($\alpha=0.9, \beta=0.5$). There are two reasons for using the parameter values to maximize the individual learning performance. The first is to show that long-term teaching can be effective even when agents have a high capacity for individual learning. The second is to reduce the computational resources and time. See the section B3 in this file for simulation results with different parameter sets. We confirmed that the main results are replicated unless the individual fails to improve the performance with individual learning. To avoid agents being trapped in a quasi-infinite loop, we limited the number of times an agent could move in one round by 1,000 steps. By repeating this reinforcement process, agents learn the value of the path to the goal and transition to the goal state without detours.

**A1. Updating the state value**

In our model, nodes have the estimated values of the state $W_{S,t}$. An agent’s arrival at the state is considered a reward. This can be seen as a process through which the agent gradually obtains more accurate estimates of value through repeated exploration and learning. If agents move from state *S* to state *S'* at time *t*, $W_{S,t}$ is updated through the following equation.

$$W_{S, t}=\left( 1-\alpha\right)W_{S, t-1}+\alpha\cdot W_{S^{'}, t-1}$$

$W_{S,t}$ is the value of the state *S* at time *t*. $\alpha$ is the learning rate for updating $W_{S,t}$, which represents the extent to which the agent learns the results obtained by the action. In this article, we set a fixed $\alpha$ value of 0.9 (see the Supplementary Information for the results of different parameter values). In this study, the state is represented by each node on the network. In the initial setting, which is at a time before the agent starts the search (*t* = 0), the values of *W* of the goal state are their reward values (the squared value of the shortest distance from the start node), and *W* of all other states is 0, which is the common initial value for all simulations in this study.

**A2. Updating the action value**

Every edge in the network has an action value $Q_{S\to B,t}$. The action value $Q_{S\to B,t}$ represents the value of action B that can be taken while in a certain state S. The agent probabilistically chooses an action according to the Q-value to choose an action that will yield a greater reward from a certain state. Agents update their estimate of $Q_{S\to B,t}$ according to the resulting reward. If action *B* is selected in the state *S* at time *t* and moved to state *S'*, $Q_{S\to B,t}$ is updated by the following formula:

$$Q_{S,B, t}=\left( 1-\alpha\right){\cdot Q}_{S\to B, t-1}+\alpha\cdot W_{S^{'}, t-1}$$

$Q_{S\to B,t}$ is the value of taking action *B* in state *S* at time *t*, $\alpha$ is the learning rate of updating $Q_{S\to B,t}$, and $\alpha$ the extent to which the agent learns the results obtained by the action. In this study, $\alpha$ is fixed at 0.9. Each node on the network represents a state, and edges refer to the action of moving to another node. Additionally, $W_{S^{'},t-1}$ is the reward that the agent obtains from the state after moving. In our model, at the point before the agent begins its search (*t* = 0), all Q values are 0, which is the common initial value for all simulations in this study.

**A3. Decision probability of action**

When the agent determines the destination node from the node they are currently at, they probabilistically determine the action (edge) they actually choose according to each Q-value and a general SoftMax rule. The probability $Pr\left( S\to B,t \right)$ of selecting action *B* in state *S* at time *t* is expressed by the following equation.

$$Pr(S\to B,t)=\frac{exp\left( Q_{S\to B,t-1} \beta\right)}{\sum_{B^{'}} \exp\left( Q_{S\to B^{'},t-1}\beta\right)}$$

$\beta$ is an inverse temperature parameter, and the small value makes the agent explore. In this article, we set a fixed $\beta$ value as 0.5 (see the Supplementary Information for the results of different parameter values). The numerator on the right-hand side is the exponential function of the Q-value of one possible action in that state, and the denominator is the sum of the exponential functions of the Q-values in all possible actions in that state. Therefore, the higher the Q-value of an action, the higher the probability of being selected. Additionally, since $Pr(S\to B,t)$ is a probability, it always assumes a value in the range of 0–1.

## **B. Supplementary figures**

## **B1. A simple example of the multi-goal network exploration**

Figure S1 depicts an example of a multi-goal network. Nodes represent states, and edges between nodes represent possible actions. When agents choose an action, they transition to an adjacent state. Edges are bi-directional, and the agent can move around the network until they reach a goal node (orange nodes). If agents reach a goal, they receive a reward. The reward is represented by the squared value of the shortest distance from the start node (upper right side of each goal node). The numbers in the nodes indicate the shortest distance from the start node (the blue node).

It was difficult to obtain large rewards with the multi-goal network we designed. When agents randomly explored the network depicted in Figure S1, 84.00% reached the goal at a distance of 1 or 2. In contrast, the chances of reaching goals at distances 3, 4, and 5 were only 7.16%, 5.32%, and 3.52%, respectively. Thus, most agents could not obtain larger rewards with this task. In our simulation (see the Results section), we designed a much more complex multi-goal network, which was more than three times the size of the network depicted in Figure S1.

To avoid agents being trapped in a quasi-infinite loop, we limited the number of times an agent could move in one round by 1,000 steps. By repeating this reinforcement process, agents learn the value of the path to the goal and transition to the goal state without detours. As agents repeat many rounds of reinforcement learning, the probability of getting an even larger reward increases. For instance, when 10,000 agents explored the network depicted in Figure S1 for 10,000 rounds, 25.46% reached the goal at a distance of 5.


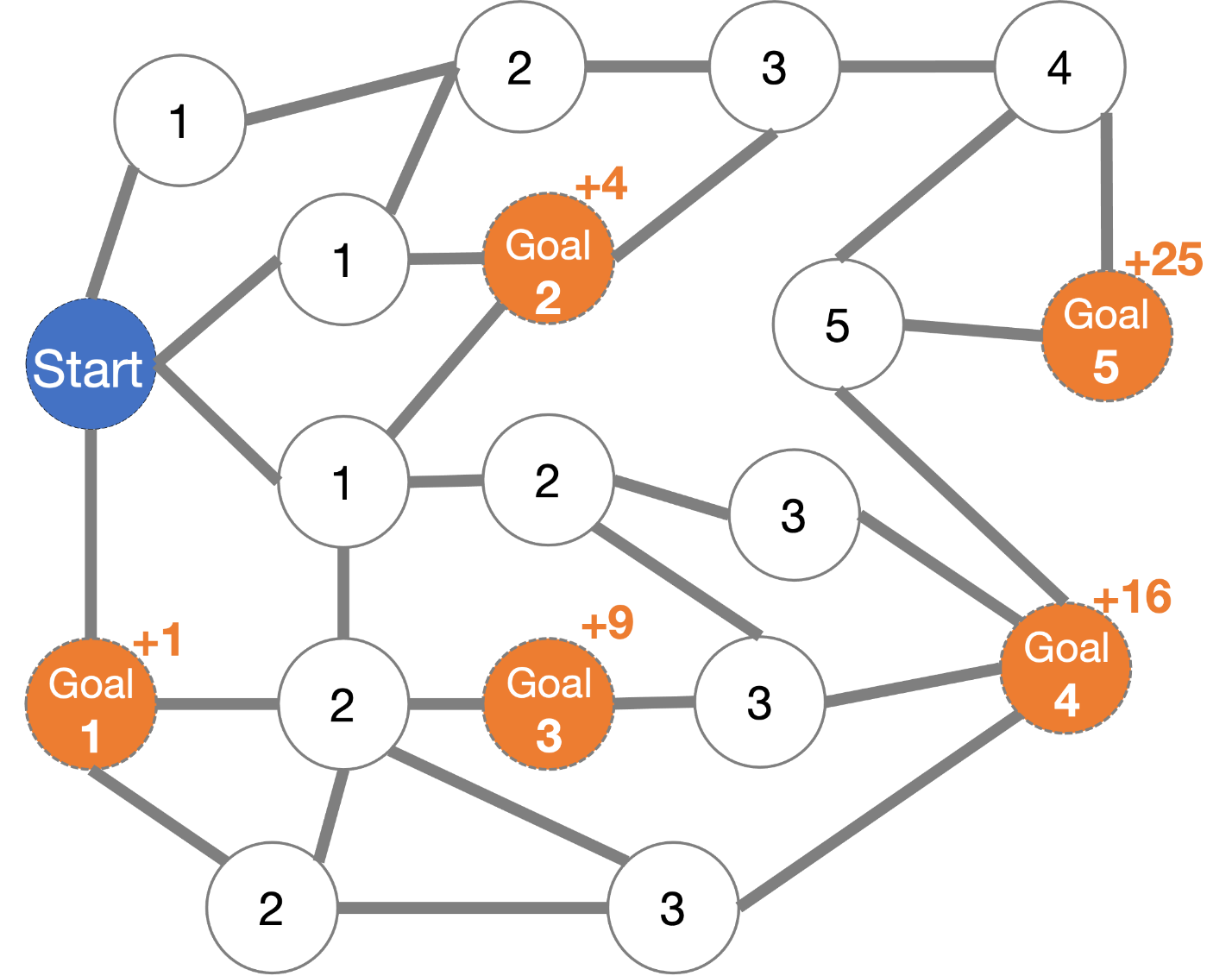


Figure S1. A simple example of the multi-goal network. The number in each node indicates the shortest distance from the starting point. The blue node represents the start node. The orange nodes represent goal nodes with rewards.

## **B2. Performance of random exploration of the network (Section “Difficulty of the task: The simulation of individual learning using the multi-goal network”)**

We ran 1,000 simulations of individual learning to test the difficulty of the task. In each simulation, an agent explored the network for only one round (i.e., random exploration without updating values). Approximately half of the agents (48.9%) reached goal 1, and another half (46.7%) reached goal 2. The number of agents who reached goals 3 and 4 were 1.8% and 2.6%, respectively. None of them reached goals 5 and 6 (Figure S2).


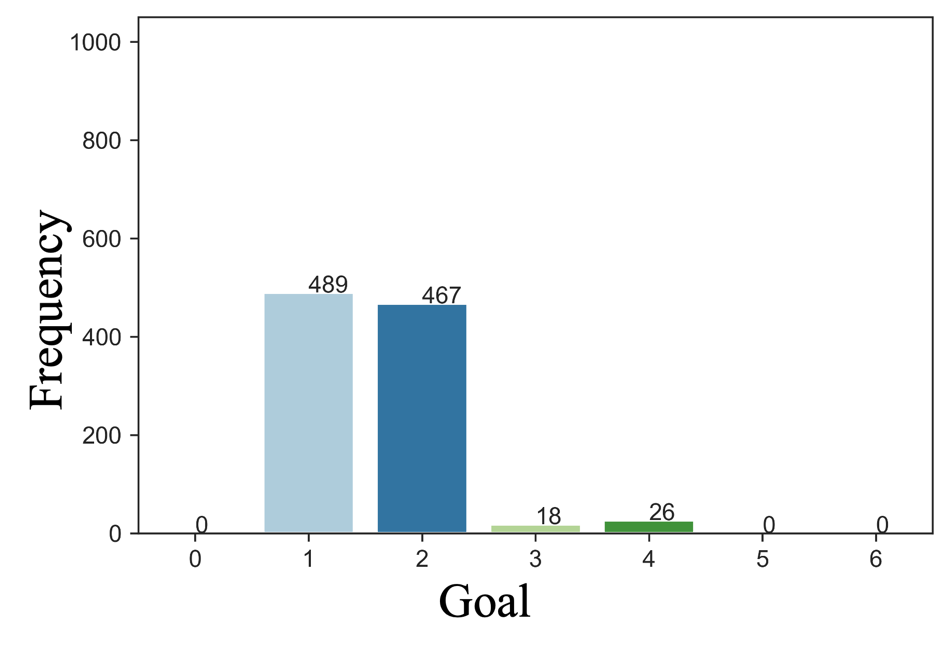


Figure S2. Frequency of the agents who acquired each reward through individual learning for only one round ($\alpha=0.9, \beta=0.5$).

## **B3. Cumulative Cultural Evolution under various learning parameters (**$\boldsymbol{\alpha}\mathbf{and}\boldsymbol{\beta}$**)**

The performance of individual learning is influenced by a combination of two parameters: learning rate and inverse temperature. The learning rate ($\alpha$) adjusts learning speed. The inverse temperature ($\beta$) weights the propensity of exploration-exploitation. We report that the results in the main text remain almost stable in response to the changes in these two parameters. Although manipulating the parameters alters the speed of individual learning, the overall results are maintained if we change the length of lifespan, *R*, accordingly.

Figure 5 in the main text depicts that the effect of long-term teaching on cumulative cultural evolution (CCE) depends on the task difficulty (determined by *R*). Here, we confirm that the results could be reproduced under three different sets of learning parameters. Like the parameter set used in the main text ($\alpha=0.9, \beta=0.5$), the first two parameter sets allow an individual to improve their performance through individual learning ($\alpha=0.5, \beta=0.5$ in Figure S3; $\alpha=0.9, \beta=1.5$ in Figure S4). In contrast, the third parameter set makes it difficult for individuals to improve their performance within a given range of *R* ($\alpha=0.6, \beta=0.2$ in Figure S5).

We illustrated the results with $\alpha=0.5, \beta=0.5$ in Figure S3. In the simulation with *R* = 10, we found that the agents were not able to increase performance within their lifetime regardless of the length of the teaching phase because we decreased the value of the learning rate parameter (Figure S3b). When the value of *R* is sufficiently large, results reported in the main text are reproduced. In the simulation with *R* = 100, we found a positive relationship between the length of teaching and performance (Figure S3d). In simulations with *R* = 400 and *R* = 5,000, where the task is even easier, the relationship between length of teaching and mean reward was reversed, which represented a negative correlation (Figures S3f and S3h). The simulation results of the immortal agent are also reproduced. Figure S3 shows that immortal agents without information loss improve their performance faster than agents who receive teaching (as shown in Figure 5 in the main text). In the simulation with *R* = 100, agents with limited lifespans can catch up to the performance of immortal agents by accumulating knowledge over generations through teaching.

Simulations with higher exploitation propensities ($\alpha=0.9, \beta=1.5$) also qualitatively replicate the results reported in the main text (Figure S4). Higher values of $\beta$ increase the agents’ possibility of being trapped in goals with lower rewards and thus reduce the variance of agent performance. Nevertheless, the effect of the value of *R* on the relationship between the length of the teaching phase and performance is reproduced. In simulations with *R* = 10, agents perform better with longer teaching phases (Figure S4b). As the value of *R* increases, such a relationship disappears (Figures S4d and S4f), and eventually, longer teaching phases lead to lower performance (Figure S4h).

When the value of learning parameter is low ($\alpha=0.6, \beta=0.2$), it is difficult to improve performance through individual learning, and CCE is also less likely to occur for ranges of *R* values used here (Figure S5). Innovation and cultural transmission are necessary for the manifestation of CCE, but this is where the former is absent.


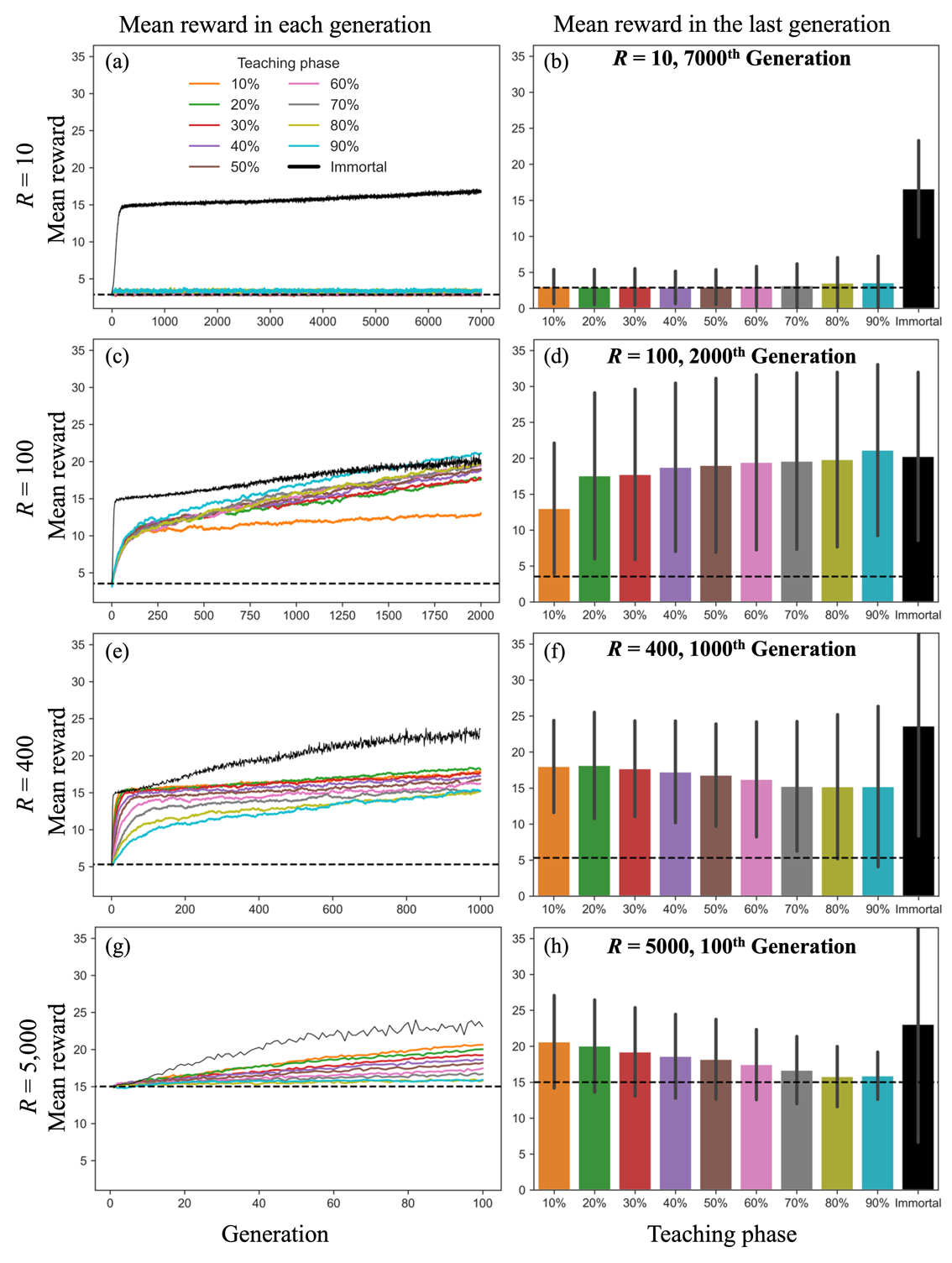


Figure S3. Comparison of the performance of immortal agents with that of agents with teaching ($\alpha=0.5, \beta=0.5$). Left column: The solid black lines represent the mean reward of 1,000 agents who have performed only individual learning corresponding with the cumulative number of rounds of the simulation with teaching. Colored lines show the mean reward in the final round of each generation with each length of teaching phase. Right column: Colored bars show the mean reward in the last generation among the length of teaching phase, and the black bar shows the mean reward of immortal agents in the final round. Error bars denote the standard deviation. Dashed lines in all figures show mean reward acquired through individual learning alone for *R* rounds.


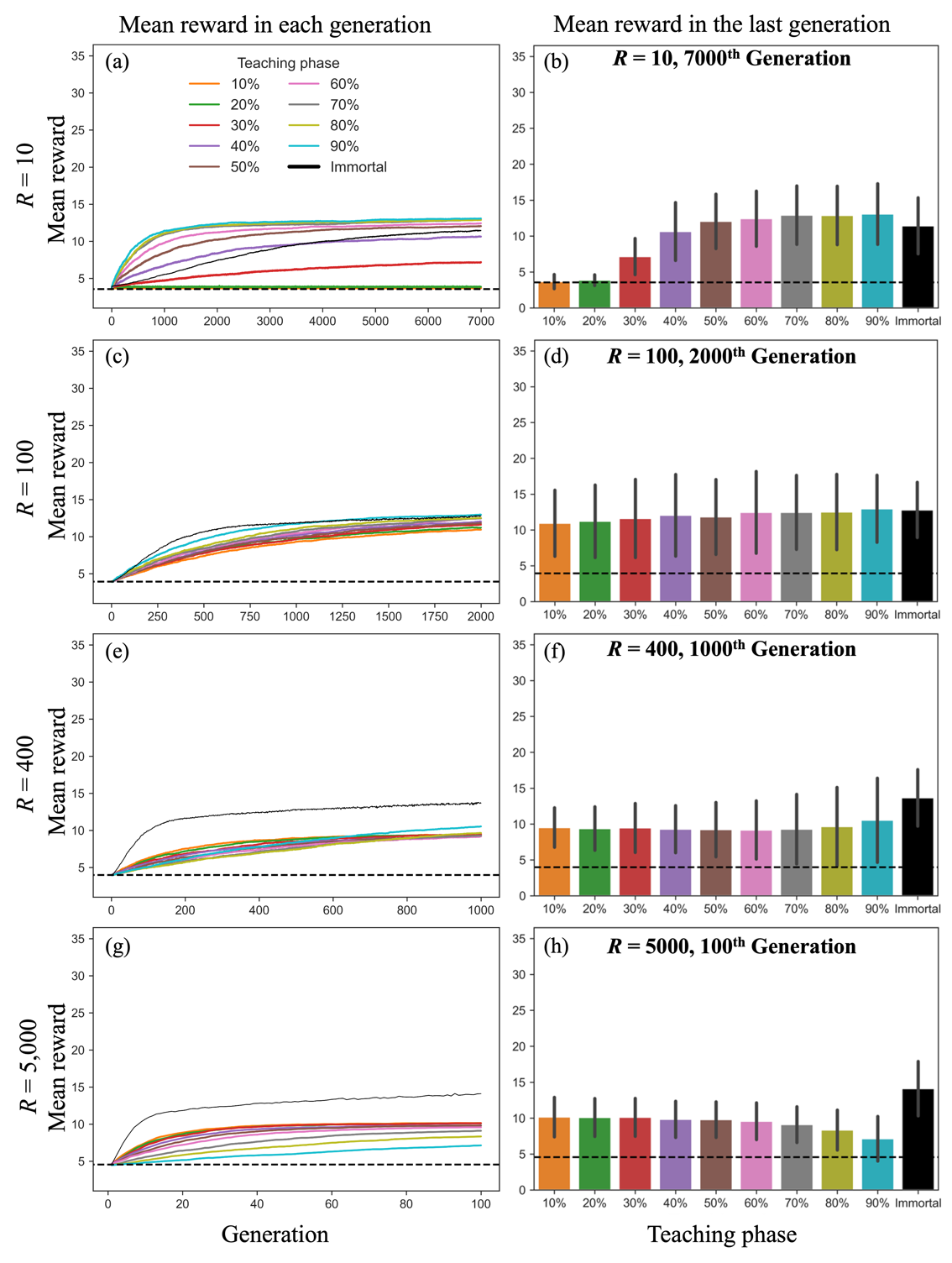


Figure S4. Comparison of the performance of immortal agents with that of agents with teaching ($\alpha=0.9, \beta=1.5$). Left column: The solid black lines represent the mean reward of 1,000 agents who have performed only individual learning corresponding with the cumulative number of rounds of the simulation with teaching. Colored lines show the mean reward in the final round of each generation with each length of teaching phase. Right column: Colored bars show the mean reward in the last generation among the length of teaching phase, and the black bar shows the mean reward of immortal agents in the final round. Error bars denote the standard deviation. Dashed lines in all figures show mean reward acquired through individual learning alone for *R* rounds.


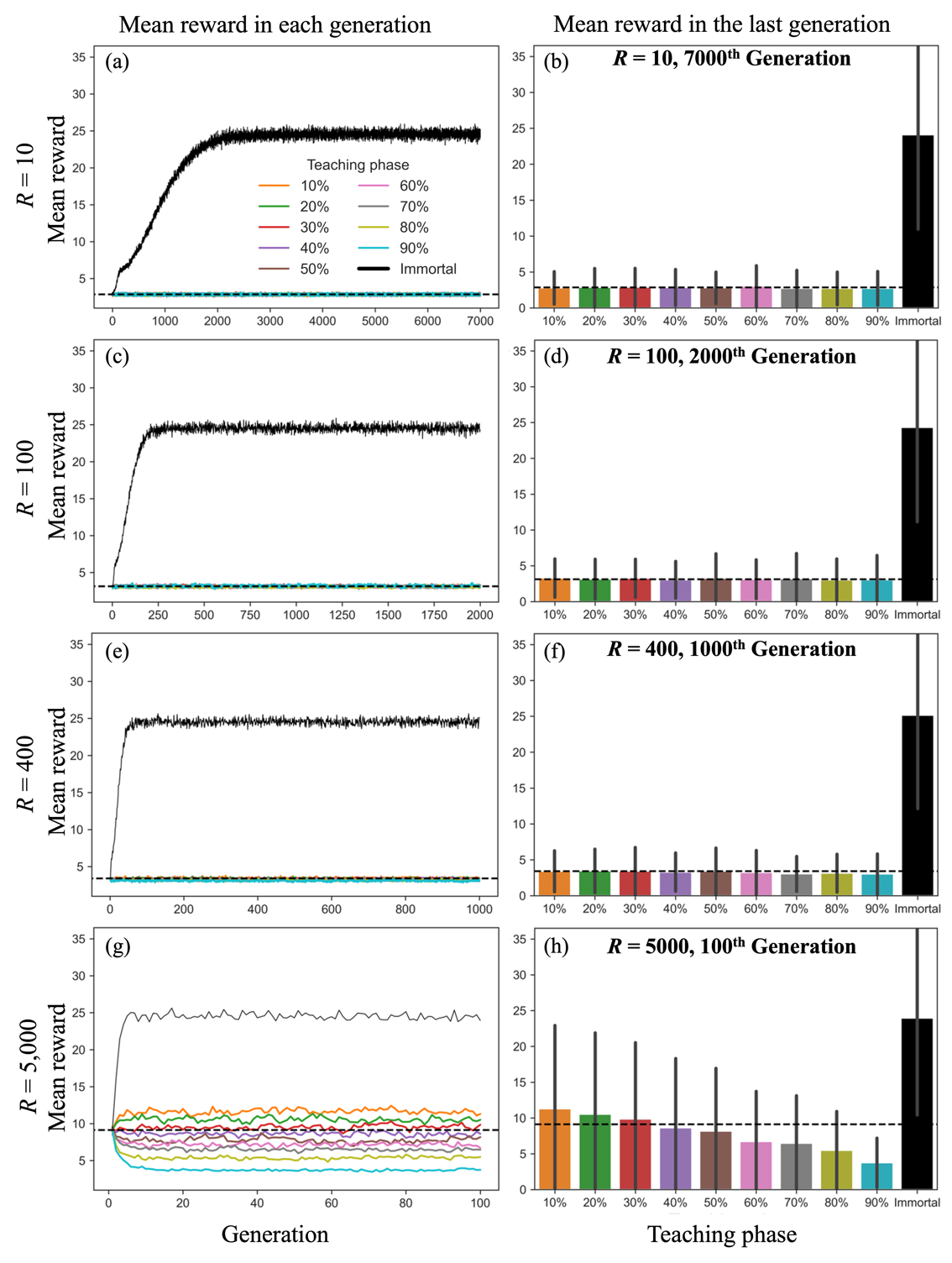


Figure S5. Comparison of the performance of immortal agents with that of agents with teaching ($\alpha=0.6, \beta=0.2$). Left column: The solid black lines represent the mean reward of 1,000 agents who have performed only individual learning corresponding with the cumulative number of rounds of the simulation with teaching. Colored lines show the mean reward in the final round of each generation with each length of teaching phase. Right column: Colored bars show the mean reward in the last generation among the length of teaching phase, and the black bar shows the mean reward of immortal agents in the final round. Error bars denote the standard deviation. Dashed lines in all figures show mean reward acquired through individual learning alone for *R* rounds.

## **B4. Results from a network with a different structure**

To confirm that the simulation results in the main text are not bounded to the specific network with three clusters, we generated a large single-cluster network and re-ran the simulation with three different reinforcement learning parameter sets: one that is used in the main text ($\alpha=0.9, \beta=0.5$) and the others examined in the previous section ($\alpha=0.5, \beta=0.5$, and $\alpha=0.9, \beta=1.5$). We followed the Erdős-Rényi model^[1]^ and constructed a huge single random network with 4,000 nodes. The average order of the network is approximately 15 (Figure S6a), which is close to the average degree of a three-cluster network. Figure S6b depicts the frequency of individual learning agents reaching each goal when 1,000 agents explored the network for 40,000 rounds. Compared to the network in the main text, the frequency of agents reaching the goal with high rewards in a short number of rounds is higher. This indicates that the task is easier.

Figure S7 illustrates the simulation results with $\alpha=0.9, \beta=0.5$—the same learning parameter settings as the main text. Under this parameter setting, the results obtained were similar to the simulation of a three-cluster network. In the simulation with *R* = 10, we observed a positive relationship between the length of teaching and performance (Figure S7b). In the simulations with *R* = 100 and *R* = 400, where the task is easier, the positive relationship disappears (Figures S7d and S7f). In the simulation with *R* = 5,000, the relationship between the length of teaching and mean reward is reversed, which denotes a negative correlation (Figure S7h). Furthermore, the agents in the simulation with *R* = 100 and *R* = 400 outperform the immortal agents regardless of the length of teaching.

Figure S8 illustrates the simulation results with $\alpha=0.5, \beta=0.5$. Under this parameter setting, there was no clear relationship between the length of the teaching phase and performance. In the simulation with *R* = 10, no CCE occurred, just like the simulation results obtained for the three-cluster network (Figure S8b). While we observed the presence of CCE, the effect of the length of the teaching phase was masked because all the agents performed very well—comparable to immortal agents—regardless of the length of the teaching phase. In the simulation with *R* = 5,000, wherein the task is much easier for the agent, we observed neither the presence of CCE nor an effect of the length of teaching because agents displayed high performance even within their lifespan (shown as black dashed lines in Figures S8g and S8h).

Figure S9 illustrates the simulation results with $\alpha=0.9, \beta=1.5$. In the simulation with *R* = 10, the positive relationship between the length of teaching and performance is reproduced (Figure S9b). On the other hand, the relationship between the length of teaching and performance remained ambiguous for *R* = 100 and *R* = 400 (Figure S9d and S9f). In the simulation with *R* = 5,000, the relationship between the length of teaching and performance showed a negative correlation (Figure S9h).


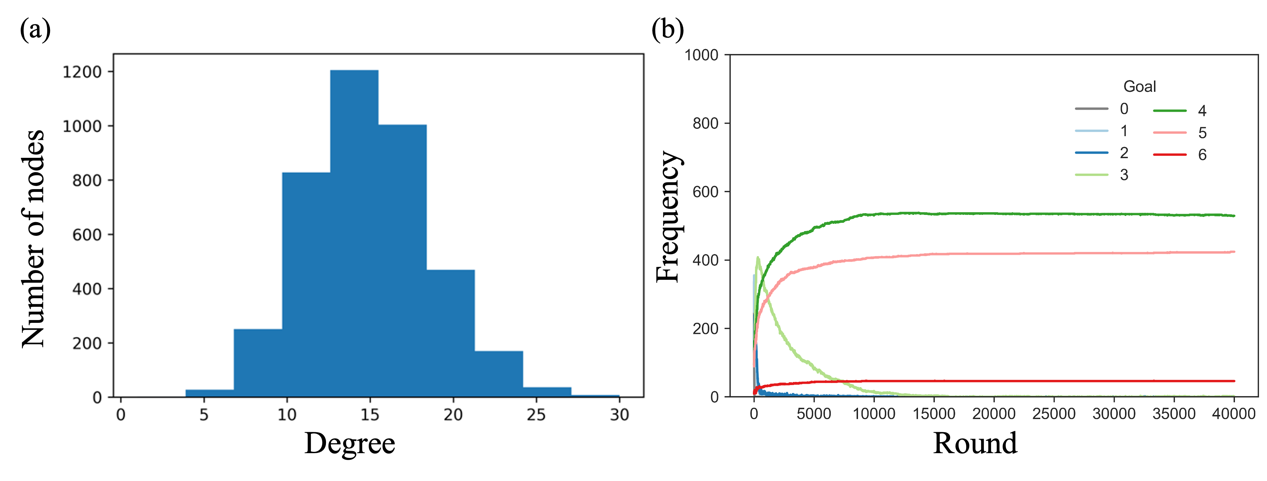


Figure S6. (a) Degree distribution of the huge single random network. (b) Frequency of the agents who reached each goal among 40,000 rounds ($\alpha=0.9, \beta=0.5$).


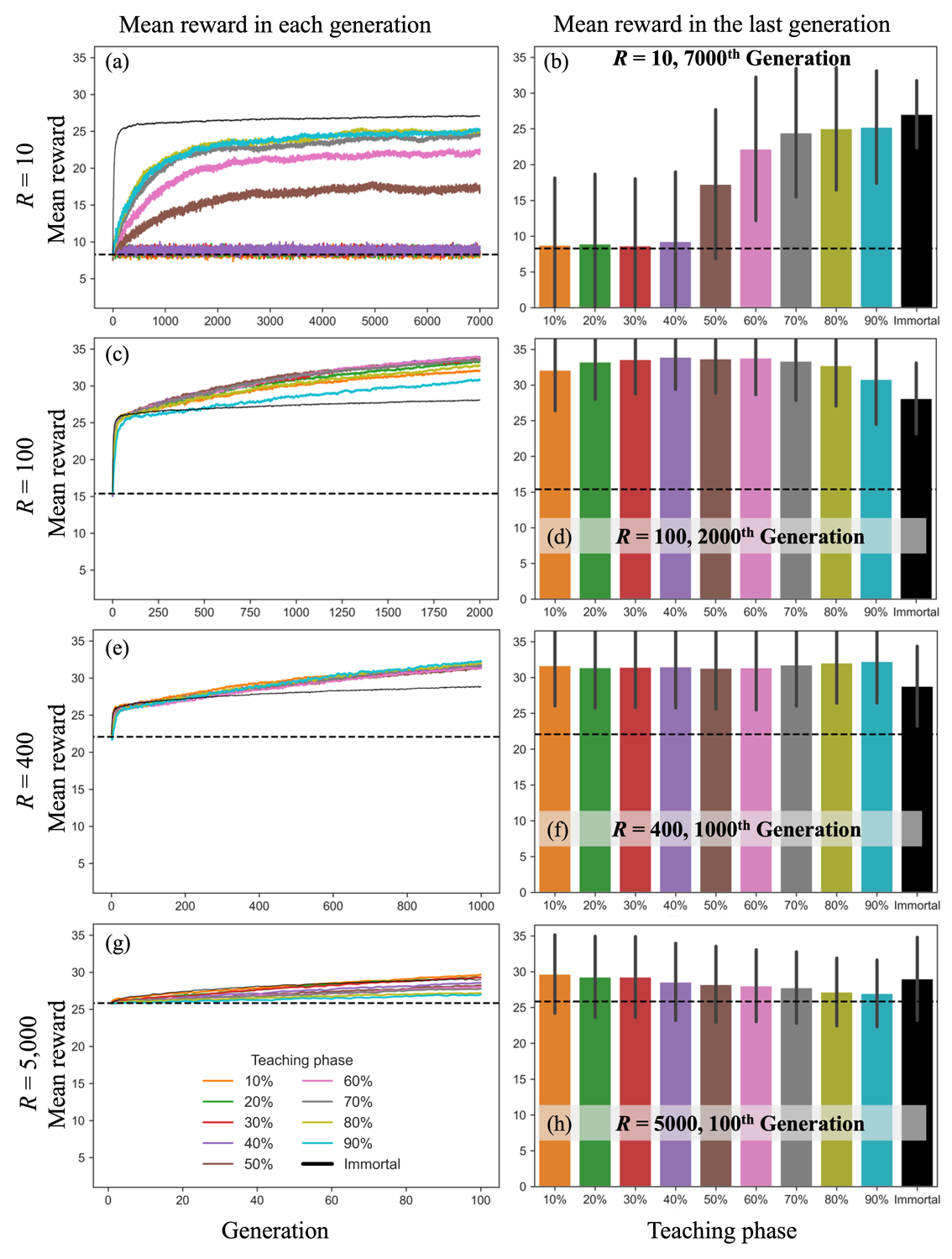


Figure S7. Comparison of the performance of immortal agents with that of agents with teaching ($\alpha=0.9, \beta=0.5$). Left column: The solid black lines represent the mean reward of 1,000 agents who have performed only individual learning corresponding with the cumulative number of rounds of the simulation with teaching. Colored lines show the mean reward in the final round of each generation with each length of teaching phase. Right column: Colored bars show the mean reward in the last generation among the length of teaching phase, and the black bar shows the mean reward of immortal agents in the final round. Error bars denote the standard deviation. Dashed lines in all figures show mean reward acquired through individual learning alone for *R* rounds.


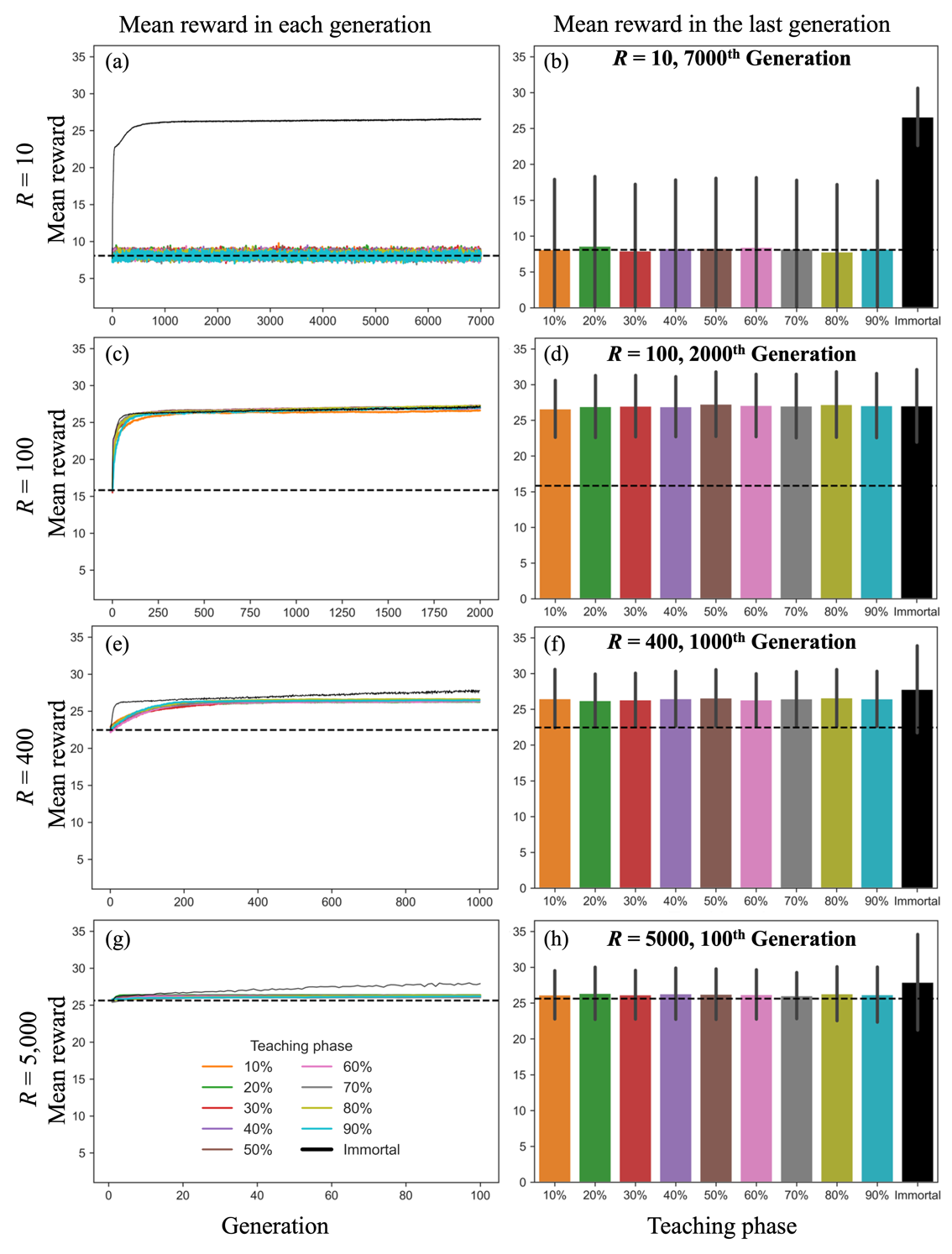


Figure S8. Comparison of the performance of immortal agents with that of agents with teaching ($\alpha=0.5, \beta=0.5$). Left column: The solid black lines represent the mean reward of 1,000 agents who have performed only individual learning corresponding with the cumulative number of rounds of the simulation with teaching. Colored lines show the mean reward in the final round of each generation with each length of teaching phase. Right column: Colored bars show the mean reward in the last generation among the length of teaching phase, and the black bar shows the mean reward of immortal agents in the final round. Error bars denote the standard deviation. Dashed lines in all figures show mean reward acquired through individual learning alone for *R* rounds.


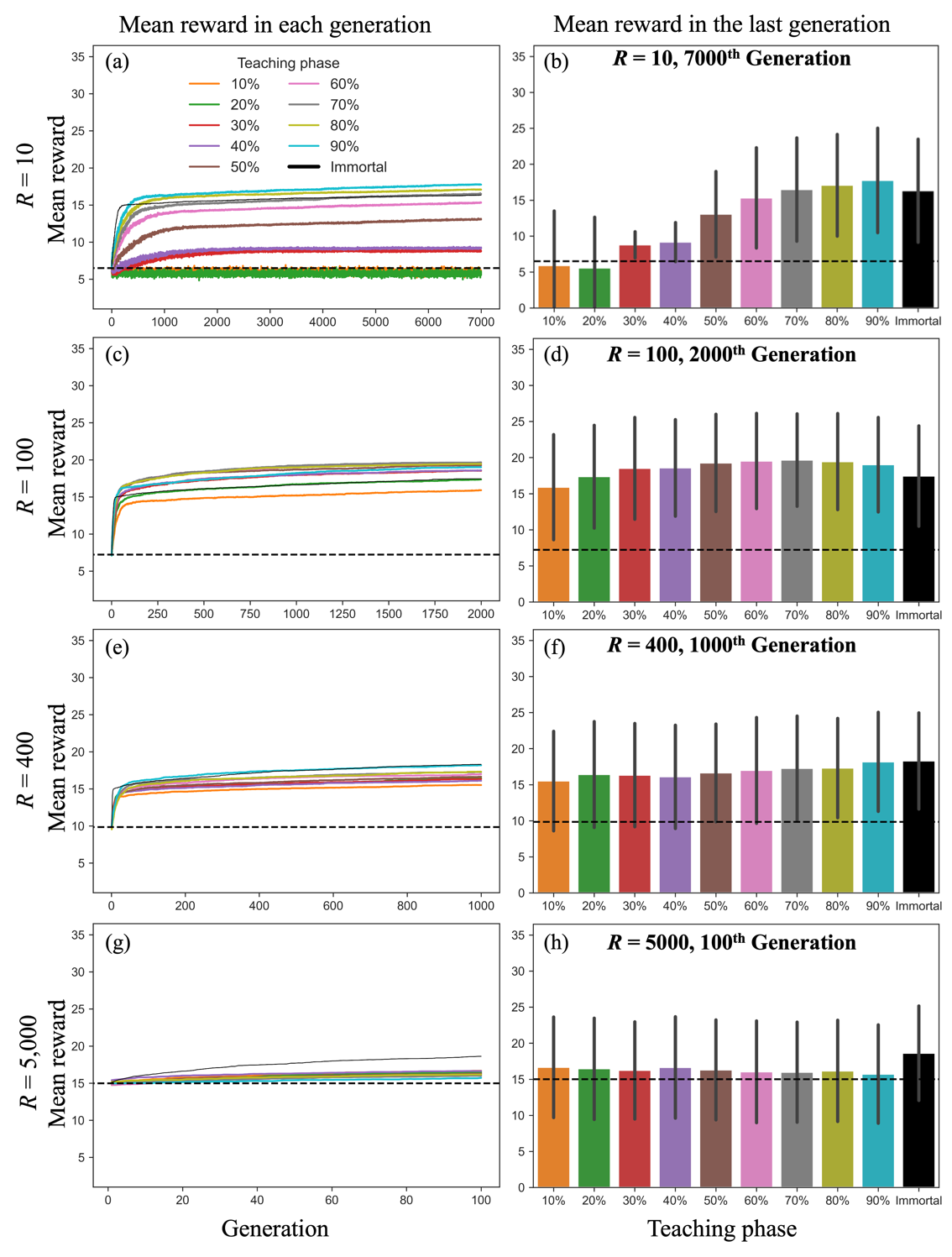


Figure S9. Comparison of the performance of immortal agents with that of agents with teaching ($\alpha=0.9, \beta=1.5$). Left column: The solid black lines represent the mean reward of 1,000 agents who have performed only individual learning corresponding with the cumulative number of rounds of the simulation with teaching. Colored lines show the mean reward in the final round of each generation with each length of teaching phase. Right column: Colored bars show the mean reward in the last generation among the length of teaching phase, and the black bar shows the mean reward of immortal agents in the final round. Error bars denote the standard deviation. Dashed lines in all figures show mean reward acquired through individual learning alone for *R* rounds.

## **B5. Results of the other teacher models**

In the simulation in the main text, the teacher agents taught the paths they took in the final round, which were probabilistically generated by applying the SoftMax rule. We also ran simulations with two alternative teaching models. It is often assumed that teachers behave differently in front of students to enhance teaching performance^[2]^. Here, we examined two different models of teachers who behave greedily by fully exploiting the Q values they have learned in their lifetime. This modified teaching model better captures the nature of teaching in that the teachers behave differently when teaching than when doing individual learning.

In the first model, after finishing the individual learning phase, the teachers generate a single path by selecting the path with the highest Q value at each state, which is analogous to applying the SoftMax rule with an infinitely positive inverse temperature value. They teach a single generated path to the agent in the next generation. We ran the simulation with the same parameter values as described in the main text ($\alpha=0.9, \beta=0.5$). Figure S10 illustrates the simulation results with the modified teaching model 1. We found that CCE is less likely to occur for the ranges of *R* values used here. In the simulation with *R* = 400 and 5,000, the short-term teaching caused slight CCE, and we can find a negative correlation between the length of the teaching phase and the performance. Because greedy behavior preferred shorter paths, the teaching in this modified teaching model 1 may have led students to goals with lower rewards.

In the second model, after finishing the individual learning phase, multiple paths (the number of which is identical to the length of teaching phase) were generated by the same greedy teacher as in the first model. When the teacher's individual learning is incomplete, which occurs when the individual learning phase length is short, multiple actions in each state tend to have the same Q value. Hence, in the second model, the teacher could probabilistically generate several different paths, even if they behave greedily. We ran the simulation with the same parameter values as described in the main text ($\alpha=0.9, \beta=0.5$). Figure S11 illustrates the simulation results with the modified teaching model 2. The same results as in the modified teaching model 1 were reproduced. Modified teaching model 2 introduced the possibility of path variation. In reality, however, there was probably little variation.

These results may indicate that a balance between exploration and faithful transmission is important. Both the final round path taught in the main text model and the greedy path taught in the modified teaching model can be considered quasi-optimal paths, but the former is the more redundant path. It may be that teaching the redundant path is more likely to result in CCE by encouraging students' exploration in individual phases. On the other hand, teaching the bare minimum may be efficient, but such teaching may inhibit exploration in the next generation.


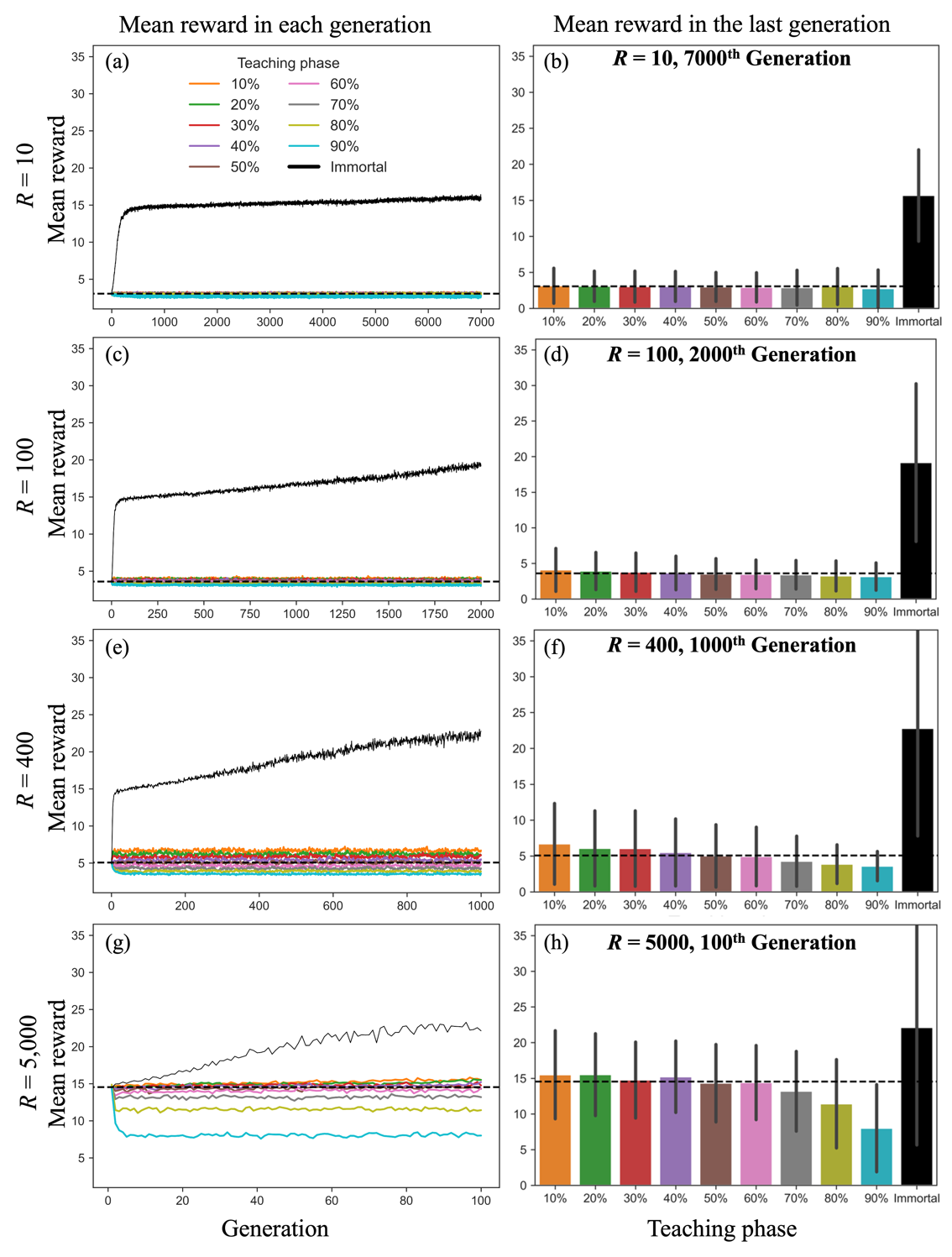


Figure S10. Comparison of the performance of immortal agents with that of agents with the modified teaching model 1 ($\alpha=0.9, \beta=0.5$). Left column: The solid black lines represent the mean reward of 1,000 agents who have performed only individual learning corresponding with the cumulative number of rounds of the simulation with teaching. Colored lines show the mean reward in the final round of each generation with each length of teaching phase. Right column: Colored bars show the mean reward in the last generation among the length of teaching phase, and the black bar shows the mean reward of immortal agents in the final round. Error bars denote the standard deviation. Dashed lines in all figures show mean reward acquired through individual learning alone for *R* rounds.


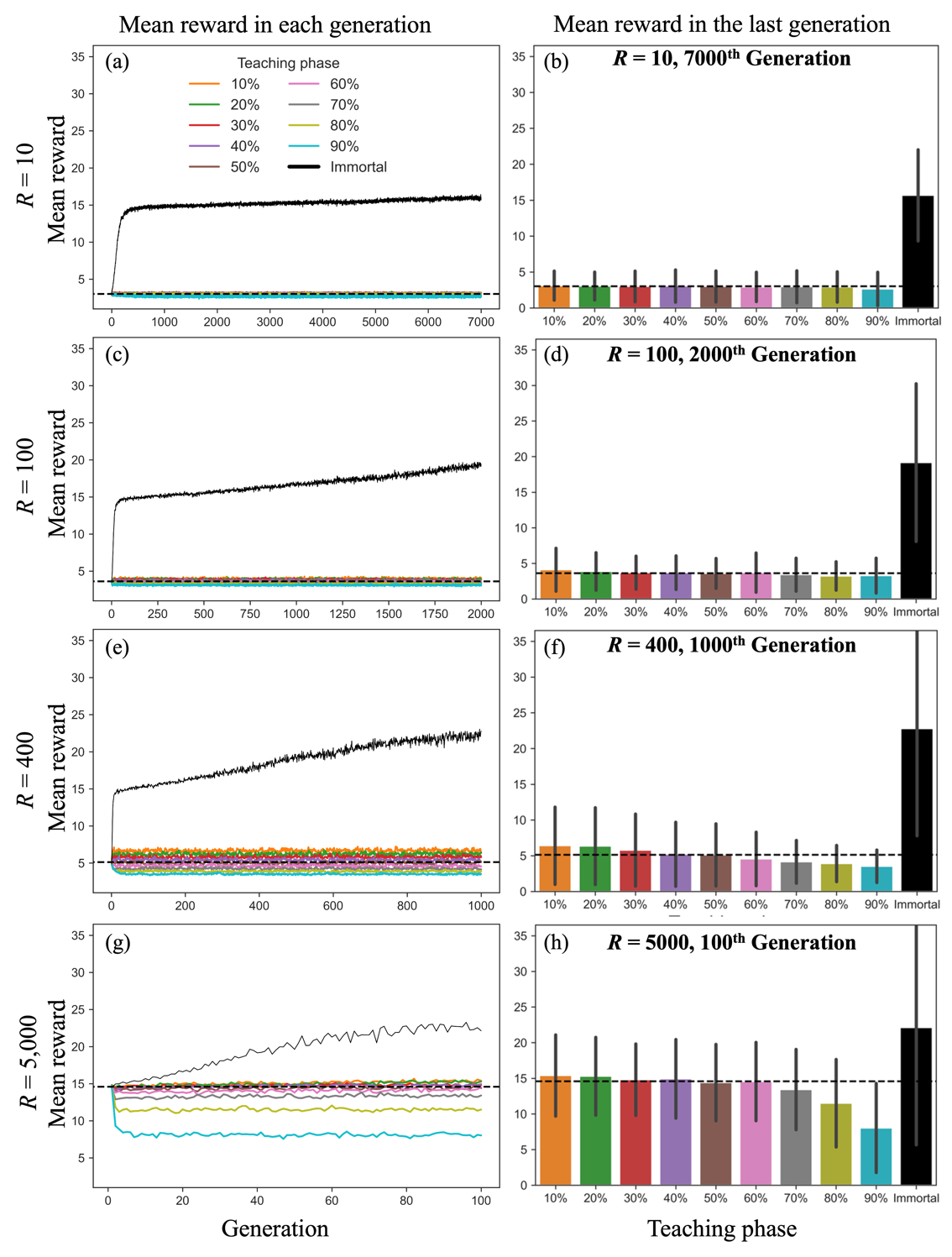


Figure S11. Comparison of the performance of immortal agents with that of agents with the modified teaching model 2 ($\alpha=0.9, \beta=0.5$). Left column: The solid black lines represent the mean reward of 1,000 agents who have performed only individual learning corresponding with the cumulative number of rounds of the simulation with teaching. Colored lines show the mean reward in the final round of each generation with each length of teaching phase. Right column: Colored bars show the mean reward in the last generation among the length of teaching phase, and the black bar shows the mean reward of immortal agents in the final round. Error bars denote the standard deviation. Dashed lines in all figures show mean reward acquired through individual learning alone for *R* rounds.

**Reference**

[1]. Erdös, P. & Rényi, A. On random graphs. *Publ. Math. Debrecen* **6**, 290–297 (1959).

[2]. Caro, T. M. & Hauser, M. D. Is there teaching in nonhuman animals? *Q. Rev. Biol.* **67**, 151-174 (1992).
